# The Role of Platelets in Diabetic Kidney Disease

**DOI:** 10.3390/ijms23158270

**Published:** 2022-07-27

**Authors:** Ukhti Jamil Rustiasari, Joris J. Roelofs

**Affiliations:** 1 Department of Pathology, Amsterdam UMC, University of Amsterdam, 1105 AZ Amsterdam, The Netherlands; u.j.rustiasari@amsterdamumc.nl; 2 Amsterdam Cardiovascular Sciences, University of Amsterdam, 1105 AZ Amsterdam, The Netherlands; 3 Department of Anatomical Pathology, Faculty of Medicine, Universitas Islam Indonesia, Yogyakarta 55584, Indonesia

**Keywords:** diabetes, diabetic kidney disease, diabetic nephropathy, platelets, platelet activation, biomarker, antiplatelet

## Abstract

Diabetic kidney disease (DKD) is among the most common microvascular complications in patients with diabetes, and it currently accounts for the majority of end-stage kidney disease cases worldwide. The pathogenesis of DKD is complex and multifactorial, including systemic and intra-renal inflammatory and coagulation processes. Activated platelets play a pivotal role in inflammation, coagulation, and fibrosis. Mounting evidence shows that platelets play a role in the pathogenesis and progression of DKD. The potentially beneficial effects of antiplatelet agents in preventing progression of DKD has been studied in animal models and clinical trials. This review summarizes the current knowledge on the role of platelets in DKD, including the potential therapeutic effects of antiplatelet therapies.

## 1. Introduction

Diabetic kidney disease (DKD) is a severe complication of diabetes mellitus that is currently the leading cause of chronic kidney disease (CKD) and end-stage kidney disease (ESKD) [1,2]. The prevalence of DKD increases in line with the rise of diabetes cases throughout the world. Diabetes is estimated to affect more than 8% of the global population (around 350 million people). Moreover, by 2040, the International Diabetes Federation has estimated that the prevalence of diabetes will increase to 642 million people [1,3,4,5]. More than 40% of people with diabetes will develop kidney disease. Currently DKD is the most common cause of ESKD in industrialized countries and a major cause of ESKD in low- and middle-income countries. Once DKD progresses to ESKD, it requires management with renal replacement therapies (dialysis and or transplantation). These therapies are associated not only with high rates of morbidity and mortality but also with a significant increase in economic burden as kidney function declines [2,3,5,6].

The etiology of DKD is complex and multifactorial. Various pathologic mechanisms have been identified, and may include factors such as metabolic abnormalities, hemodynamic changes, tubulo-toxicity, oxidative stress, and induction of inflammatory and coagulation responses, which in concert lead to irreversible damage and renal fibrosis [7]. Among these mechanisms, the derailing of inflammation and coagulation responses appears to be the most plausible one for the development and progression of DKD [7,8,9].

Platelets have been shown to play a part in the pathogenesis of glomerular disease through various mechanisms. Besides their primary physiological role in hemostasis, platelets play a pivotal role in inflammatory processes. Platelet activation is a well-known phenomenon in patients with various type of renal diseases, and research has shown that platelets are also involved in the pathogenesis of DKD [10]. Multiple studies offer evidence of enhanced activation of platelets in patients with diabetes mellitus. The increased platelet hyperreactivity in diabetes and DKD orchestrates the release of multiple chemical substances and proteins that are stored in α-granules, dense granules, and lysosomal granules, including molecules that act as pro-inflammatory mediators, pro-fibrotic mediators, growth factors, and vasoactive mediators contributing to the pathophysiology of DKD [11,12,13,14]. In this review, we discuss the current evidence for the role of platelets in inflammatory, coagulation, and fibrosis processes in DKD, and an overview of current antiplatelet drugs intended for the treatment of DKD.

## 2. Diabetic Kidney Disease

DKD, one of the major microvascular complications of diabetes mellitus, is characterized by structural and functional changes. The most characteristic features of DKD occur in the glomeruli, including thickening of the basement membrane, the earliest electron microscopical lesion observed in patients with DKD, deposition of extracellular matrix (ECM) in the mesangial area, and the most commonly observed histopathological lesion by light microscopy, either in diffuse or nodular pattern, known as nodular glomerulosclerosis (Kimmelstiel–Wilson nodules). Glomerular lesions occur together with specific vascular lesions including arteriolar hyalinosis as a result of the accumulation of hyaline material, the product of plasma protein exudation, which typically occurs in both afferent and efferent arterioles architecture [1,15,16,17,18].

The pathological changes in the glomerular structure in DKD have been clearly demonstrated. Many recent studies have shown that the proximal renal tubule plays an important role in the pathogenesis of DKD as well. A growing body of evidence shows that renal tubular damage is not only secondary to glomerular damage, but a promoting factor in DKD in itself, in patients with or without proteinuria [19]. Indeed, urinary levels of tubular injury marker KIM-1 are strongly associated with progressive renal decline, independent from markers of glomerular damage in non-proteinuric T1DM patients [20]. Diverse mechanisms, among which hypoxia, mitochondrial dysfunction, activation of innate immune mechanisms, and autophagy contribute to primary proximal tubular injury, already in very early stages of DKD [19,21]. Damage to the proximal renal tubule causes an inflammatory response that orchestrates tubulointerstitial damage [21,22].

Eventually, progression of DKD will lead to nonspecific tubulo-interstitial changes, including tubular atrophy, accumulation of activated myofibroblasts, collagen, inflammatory cells, and loss of capillary architecture [1,15,16,17,18]. These histologic lesions are indistinguishable in type 1 diabetes mellitus (T1DM) and type 2 diabetes mellitus (T2DM), which also show overlap in the occurrence of renal complications [15,16].

Clinically, DKD is characterized by progressive kidney damage that is reflected by increasing albuminuria (more than 300 mg/24 h (macroalbuminuria) or >200 µg/min) or an albumin creatinine ratio >300 mg/g creatinine, impairment in renal function (decline in glomerular filtration rate/GFR) with estimated GFR (eGFR) <60 mL/min/1.73 m^2^, elevated blood pressure, and excess morbidity and mortality caused by cardiovascular complications [23]. To resolve the discrepancy between the existing classification of DKD and the current classification of CKD Stages, the Joint Committee on DKD revised its classification of DKD in 2014 (Table 1) [24].

## 3. Mechanisms of Tissue Damage in DKD

### 3.1. Renal Inflammation in DKD

Poor glycemic control is one of the major risk factors for the development of DKD. This condition leads to a complex series of pathophysiological alterations related to dysregulation of glucose metabolism. Various mechanisms related to the hyperglycemic state are involved in the development of DKD, which include metabolic disorders, hemodynamic alterations (e.g., hyperfiltration and hyperperfusion), podocyte injury caused by hyperglycemia-induced ROS generation, autophagy and apoptosis, activation of the renin-angiotensin system, accumulation of advanced glycation end products (AGEs), polyol pathway activation, and abnormal PKC activation [17,25]. However, these mechanisms are not sufficient to explain all the pathophysiological changes observed in diseased kidneys. Other metabolic factors, such as increased free fatty acid levels and changes in adiponectin, also contribute to metabolic imbalance and disease initiation [17]. These metabolic disturbances are closely linked to a low-grade chronic inflammatory state.

Chronic inflammation plays an important role in the development of DKD. Traditionally, DKD has been considered a non-immune, degenerative glomerular disease. The importance of inflammation in the pathogenesis of DKD was first reported by Bohle et al. [26], who in 1991 described the presence of monocytes, macrophages, T-cells, and fibroblasts associated with the tubulointerstitial changes seen in DKD. By now, a broad variety of inflammatory factors have been implicated in DKD.

The infiltration of inflammatory cells into the kidney is orchestrated by pro-inflammatory cytokines, chemokines, adhesion molecules, adipokines, Toll-like receptors, and different signs of endothelial dysfunction (Table 2) [8,27,28,29,30,31,32,33].

Several components of the diabetic environment, such as hyperglycemia, the renin-angiotensin system and oxidative stress, can activate the inflammatory process in the kidney, which in turn can release harmful molecules, such as pro-inflammatory cytokines and reactive oxygen species [32]. In DKD, the inflammatory state is further enhanced by induction of various pro-inflammatory cytokines (IL-6, IL-8, IL-10, TNF- α), chemokines (CCL2/C-C motif ligand 2), and adhesion molecules (ICAM-1 and VCAM-1), which stimulate transmigration of macrophages and T cells from the circulation to the interstitium [27]. The investigation of genetic variants in the meta-analysis by Nazir et al. [34] supports a role for inflammatory cytokine pathways in the pathogenesis of DKD.

### 3.2. Coagulation and Hemostasis in DKD

Diabetes is associated with a hypercoagulable state caused by an imbalance between hemostatic factors in plasma, and at the endothelial cell surface [35,36,37]. Hyperglycemia seems to contribute to the hypercoagulable state in diabetes by altering the glycation of numerous components and through osmotic effects on platelets that increase reactivity. In addition, insulin resistance, compensatory hyperinsulinemia, diverse cytokines, and metabolic derangements that are associated with and involved in the pathogenesis of insulin resistance also contribute to the hypercoagulability [37].

Increased blood coagulation has been shown in diabetics, a phenomenon that is more pronounced in the presence of nephropathy. The plasma levels of many clotting factors including fibrinogen, tissue factor, coagulation factors Va, VII, VIII, IX, Xa, XI, XII, thrombomodulin, kallikrein, and von Willebrand factor (vWF) are elevated in diabetes. In addition, the plasma levels of natural anticoagulants, such as activated protein C (APC) and protein S, are decreased [35,36,37].

A study by Astrug et al. in DKD patients indicated that increased coagulation manifested in changes of thrombocyte adhesion, in partial thromboplastin time (PTT), recalcification, and heparin-recalcification time [38]. Coagulation factor XII also plays a role in diabetes. FXII signaling via the urokinase-type plasminogen activator receptor (uPAR) triggers inflammasome activation (cleaved Casp-1 and IL-1β, NLRP3 expression) and renal damage in diabetic mice. Albuminuria, tubulointerstitial fibrosis, and glomerular damage were ameliorated in diabetic FXII-/- mice compared to diabetic Wt mice [39].

Several studies also revealed that fibrinolytic system abnormalities contribute to the development of diabetic microvascular and macrovascular complications. Hypofibrinolysis reduces the expression of the tissue plasminogen activator (t-PA) and increases plasminogen activator inhibitor-1, which leads to increased coagulation [35,37,40]. Indeed, the plasma levels of fibrinogen, antithrombin III (AT III), PAI-1, vWF activity, and the prothrombin time were found to be significantly increased in T2DM patients compared to healthy subjects [35]. Plasma PAI-1 levels and factor V activity were increased in diabetic patients with microvascular complications and plasma PAI-1 levels and factor VII activity were significantly higher in diabetic patients with nephropathy than in diabetic patients without nephropathy [35]. A comparison of diabetic patients with and without DKD showed significant difference in fibrinogen and PAI-1 levels [40]. DKD is also associated with high plasma homocysteine levels [41] and elevated protease-activated receptors 1 (PAR1) and PAR2, which are platelet thrombin receptors [42,43], implying another procoagulant and prothrombotic role in the DKD patients.

### 3.3. Fibrosis in DKD

Renal fibrosis is a final common pathway for almost all forms of kidney disease that progress to end-stage renal failure, including DKD [25,44]. Glomerular fibrosis in DKD manifest itself as a diffuse or nodular fibrosis (Kimmelstiel–Wilson nodule) and subsequent glomerulosclerosis. This phenomenon contributes to the occlusion of the glomerular capillaries and causes further progressive tissue damage [45,46]. Scar tissue changes are due to the proliferation of mesangial cells and accumulation of excessive matrix protein (collagen types I, III, and IV and fibronectin) produced by mesangial cells, accompanied by a decrease in the matrix protein degradation process by mesangial matrix metalloproteinases [44,46]. Fibrotic changes also include tubulointerstitial fibrosis, and in the arterial vasculature, which causes arteriosclerosis, as well as microvascular hyalinosis [44,47]. The accumulation of fibrotic tissue results in anatomic disarray and renal function impairment.

Fibrogenesis can be triggered by a variety of stimuli. Hyperglycemia or diabetic conditions are known to trigger inflammatory conditions and pro-fibrotic reactions [45]. High-glucose concentrations induce specific cellular effects, which affect many types of kidney cellular elements responsible for producing ECM that play important roles in the evolution of diabetic glomerulosclerosis, including glomerular endothelial cells, smooth muscle cells, mesangial cells, podocytes, epithelial cells of the tubular and collecting duct system, inflammatory cells, and fibroblasts [7,46,48,49]. Hyperglycemia directly alters mesangial cell function via non-enzymatic glycosylation that generates AGEs. Hyperglycemia leads to the generation of reactive oxygen species, increased flux through the hexosamine pathway, acceleration of the polyol pathway, and increased of oxidative stress. It may also increase the secretion of renin from the kidney, leading to generation of angiotensin II [49,50,51]. Hyperglycemia also inhibits production of nitric oxide (NO) by blocking eNOS (endothelial NO synthase) activation and increasing the production of reactive oxygen species (ROS). Excess amounts of ROS modulate activation of other signaling molecules, such as protein kinase C (PKC), various cytokines and transcription factors that eventually cause increased expression of ECM genes with progression to fibrosis and end-stage renal disease [52,53].

Chronic or poorly controlled hyperglycemia increases the production of several profibrotic factors such as transforming growth factor-β (TGF-β), platelet-derived growth factor (PDGF), and connective tissue growth factor/CCN family member 2 (CTGF/CCN2) and contributes to fibrotic connective tissue accumulation characterized by elevated fibronectin and collagen levels [51]. TGF-β has been recognized as an important mediator in the renal fibrosis process by stimulating signaling pathways that play a role in excessive ECM formation [54]. Hyperglycemia activates TGF-β via activation of glucose transporters (GLUT), angiotensin II, and PDGF. Activated TGF-β leads to glomerular basement membrane (GBM) thickening and glomerulosclerosis through activation of CTGF and vascular endothelial growth factor (VEGF) [51]. PDGF-B and CTGF may play a role in the development and progression of glomerulosclerosis and tubulointerstitial fibrosis. They act as growth factors, and have been identified as downstream mediators of TGF-β1, and have been shown to be overexpressed in experimental and human DKD [44,47,54,55,56].

In vitro and in vivo studies have shown that several other growth factors such as basic fibroblast growth factor (bFGF), hepatic growth factor (HGF), VEGF, and epidermal growth factor (EGF) are upregulated in DKD. These growth factors are involved in the morphological and hemodynamic changes characteristic of a diabetic kidney, including renal fibrosis, through signaling pathways that activate and ultimately regulate transcription factors affecting glomerular ECM accumulation [57,58,59,60].

Fibroblasts are known to be the main effector cells of fibrosis. The cause of renal fibrosis in diabetic nephropathy is widely believed to be phenotypic switching of fibroblasts to an activated state. When activated, they serve as primary collagen-producing cells and contribute to ECM accumulation [61,62]. During fibrosis, activated fibroblasts can also arise from non-fibroblast cells, such as mesangial cells, tubular epithelial cells, and bone marrow-derived progenitor cells [62,63]. Experimental and clinical studies of DKD indicate that phenotypic changes of mesangial and epithelial cells result in myofibroblast transdifferentiation (MFT) and epithelial-mesenchymal transition (EMT). These processes are induced by cytokine TGF-β, AGEs products generation, CTGF, and hemodynamic forces, resulting in reprogramming of differentiated kidney cells to secrete and accumulate ECM [62]. In a mouse model of DKD, Zeisberg et al. [63] demonstrated that endothelial-to-mesenchymal transition (EndMT) also plays a role. They found an increase of both fibroblast-specific protein 1 (FSP1)-positive fibroblasts and of α-smooth muscle actin (αSMA)–positive fibroblasts, which also exhibit CD31 positivity, revealing that these fibroblasts are likely of endothelial origin.

Podocytes are also involved in the glomerulosclerosis process. Lee and Kalluri (2010) showed decreased podocyte numbers in diabetic and non-diabetic glomerular diseases, which results in proteinuria and glomerulosclerosis [64]. AGE-RAGE interaction also plays a role in the sclerosis mechanism involving podocytes. RAGE, a receptor for AGEs, displays enhanced expression in podocytes from human diabetic kidney [65] and from diabetic mice [66]. The activation of RAGE contributed to the increased VEGF level, enhanced recruitment of mononuclear phagocytes in the diabetic glomerulus, and TGF-β that lead to the expansion of mesangial matrix, which converge to cause albuminuria and glomerulosclerosis [66].

## 4. The Function of Normal Platelets: Hemostasis and Thrombosis

There is general agreement that in 1882 Giulio Bizzozzero was the first to describe blood platelets as ‘spherules piastrine’ (little plates): small cell fragments that clumped together at an injured blood vessel site. He also demonstrated the role of platelets in hemostasis and in promoting experimental thrombosis and he provided the first description of platelet–leukocyte interactions [67,68].

Platelets are small anucleate cells in the circulation with a stable discoid shape in the blood circulate, remaining near to the blood vessel wall where they can quickly respond to any damage [69]. Platelets have many unique structural features that facilitate their contributions to thrombus formation. They are metabolically active cells and contain a large variety of cellular organelles such as mitochondria, endoplasmic reticulum, and Golgi apparatus. Platelets do not contain DNA, but mRNA, hence they can synthesize a limited amount of proteins. They also have a variety of surface receptors, adhesion molecules, and contain a large number of granules [70,71].

The platelet membrane is rich in a variety of glycoproteins (GP) that bind agonists to activate platelets and function primarily as adhesive molecules [72]. Platelets contain three major types of secretory organelles: α-granules, dense bodies (δ-granules), and lysosomes. α-granules are the most numerous of the organelles in platelets. They contain many proteins, including those synthesized by the megakaryocytes, platelet-selective proteins, including coagulation factors (factor V, protein S, and factors XI and XIII), adhesion molecules (thrombospondin, P-selectin, PECAM-1, and vWF), proteins synthesized in other cells and taken up by platelets, such as fibrinogen, mitogenic factors (PDGF, TGF-β, and EGF), angiogenic factors (VEGF), and chemokines. Human platelet dense bodies (DB) are smaller than the α granules, are fewer in number, and have high morphological variability. DB are rich in adenine nucleotides, including ATP and adenosine diphosphate (ADP), bioactive amines (serotonin, histamine), calcium, and magnesium. Human platelets contain few lysosomes. Platelet lysosomes may serve as an endosomal digestion compartment in platelets. Lysosomes release glycosidases, proteases, and bactericidal enzymes such as β-glucuronidase, elastase, and collagenase [73,74].

### Platelets in Hemostasis and Thrombosis

Under normal physiological conditions, platelets do not interact with the intact vessel wall. However, upon tissue trauma, platelets can quickly anchor to the subendothelial surface as the primary target site of platelet activation. Vessel damage results in a decrease in the inhibitors of platelet function in intact endothelium (the production of nitric oxide and prostacyclin and also the loss of CD39, which functions to break down ADP in the intact state). The exposure of ECM proteins such as collagen to the circulation also allows the initial recruitment of platelets [72,75,76,77].

The subendothelial ECM contains several adhesive macromolecules such as collagen, vWF, laminin, fibronectin, and thrombospondin, which serve as ligands for different platelets membrane glycoproteins [72,75,76,77,78]. Platelets adhere to various components of the subendothelium. Following this initial tethering of the platelet to the vessel wall, subsequent firm adhesion results in signal transduction within the platelet, including the rearrangement of the cytoskeleton, increased intracellular calcium, and granule release [75,79,80]. The resting platelet reorganizes its cytoskeleton and assembles new actin filaments to change its shape from a resting discoid form to an activated state with numerous pseudopodia. It also releases its granule contents, such as ADP and thromboxane A2 (TXA2), which act as platelet activators. They enhance cell activation and activate other circulating platelets. At sites of vascular injury, platelets thus adhere to each other, and form aggregates known as platelet plugs (the first wave of hemostasis) [14,72,76,77,78].

Platelets also contribute to the coagulation pathway (the second wave of hemostasis), by generating cell-based thrombin generation, which markedly amplifies the blood coagulation cascade [81]. This second phase is characterized by clot stabilization that is due to platelet mass consolidation through platelet retraction mediated by actin/myosin. At this stage, thrombin not only activates platelets, but also converts fibrinogen to fibrin [75,82]. This process is triggered by the presence of pro-coagulant factors (e.g., fibrinogen, factor V, and von Willebrand factor), which are secreted by platelets, by tissue factors (extrinsic pathways), and by contact activation (intrinsic pathways) of the coagulation system. When Factor Xa is formed, it converts prothrombin into thrombin, which induces fibrin formation. The platelet membrane provides a surface for coagulation factors. During platelet activation, platelet membrane phospholipids become negatively charged, which activates the surface that harbors the coagulation factors (FV, FVIIIa, FIXa, and FX). This not only triggers them to generate thrombin, it also activates platelets and contributes to the coagulation cascade [14,77,81]. The fibrin mesh that is formed then binds to the platelets and contributes in their attachment to damaged blood vessels. This attachment is mediated by the binding of glycoprotein receptors on platelets to fibrin and through interactions with other adhesive proteins such as thrombospondin, fibronectin, and vitronectin [77].

## 5. Platelet Dysfunction in Diabetic Condition

Hyperglycemia contributes to many complications of diabetes mellitus diabetes, including nephropathy. Hyperglycemic conditions are associated with hypercoagulability; they contribute to changes in the glycation of various components and exert an osmotic effect on platelets that leads to increased platelet reactivity [37].

The hypercoagulable state in diabetes may also be the result of the imbalance between circulating coagulation factors and the endothelial cell surface [36]. Diabetes mellitus is associated with hypercoagulable and inflammatory conditions, which strongly correlate with platelet activation and reactivity. This platelet hyperreactivity is characterized by increased activation, adhesion, and aggregation because of the dysregulation of several signaling pathways. Lim et al. have confirmed higher levels of adhesion molecules, soluble CD40 ligand (sCD40L), and soluble P-selectin (sP-sel, an index of platelet activation) in diabetic patients compared with normal controls. They also demonstrated strong correlations between sCD40L and both proinflammatory cytokine IL-6 and tissue factor as an initiator of coagulation [83].

Platelet hyperreactivity in diabetic patients is not only related to hyperglycemia, it is also associated with various metabolic conditions commonly found in diabetes mellitus, such as insulin resistance, obesity, and dyslipidemia, that contribute to increased systemic inflammation and oxidative stress [26]. An overproduction of reactive oxygen and nitrogen species and potent radicals, such as hydrogen peroxide and superoxide anion, which can directly cause platelet activation, have been reported in patients with T2DM. Oxidative stress directly affects platelet reactivity by enhancing intra-platelet calcium release upon platelet activation, which amplifies the response of platelet aggregation. The ROS increase the rate of accumulation of advanced glycation end products (AGEs) by enhancing the interaction of sugars with proteins during recurrent episodes of hyperglycemia. The AGEs products can interact with their receptors (RAGEs) on the endothelium, thus inducing endothelial dysfunction and an inflammatory response [84]. Endothelial dysfunction leads to decreased production of nitric oxide (NO) and prostacyclin (PGI2), two inhibitors of platelet aggregation. Hence, the reduction in NO and PGI2 may also contributes to platelet hyper-reactivity [84,85].

Increased F2 isoprostane production (prostaglandin-like compound), as a result of oxidative stress, may also lead to platelet hyperactivity by enhancing platelet response to agonist-induced platelet adhesion and aggregation, and by increasing the signaling of platelet receptors [13,86]. Furthermore, both oxidative stress and inflammation are also associated with an accelerated turnover of platelets, as indicated by the presence of immature circulating platelets. These large platelets are inherently hyperreactive and less responsive to antiplatelet therapy [84].

Platelet size associates with platelet function, and larger platelets are more active than smaller platelets. Larger platelets are metabolically and enzymatically more active, contain denser granules, secrete more serotonin and β-thromboglobulin, and produce more TXA2 and adhesion molecules than smaller platelets. Mean platelet volume (MPV) blood test measures the average size of platelets and is considered a marker of platelet activation [13,85,87,88].

T2DM patients were reported to have a much higher MPV than the healthy control group, this had an effect on the increase in the amount of α-granule content in the cytoplasm as a sign of dysfunction of the megakaryocyte-platelet system. Increased platelet volume may play a role in the development of vasculopathies and complications in diabetes mellitus. The MPV value also increases along with the development of the DKD stage. [13,85,87]. The study of Bavbek et al. [88] including 114 diabetic patients and 31 healthy controls, of which 75 (66%) diabetic patients indicated nephropathy, also showed that MPV was significantly greater in DKD patients compared to controls. Therefore, platelets can be an important entity for estimating vascular risk in T2DM.

## 6. Role of Platelet in Pathogenesis of DKD

Platelets have been shown to play a role in the pathogenesis of glomerular disease through various mechanisms. Besides having an important physiology in preventing blood loss when the vasculature is damaged, platelets also play an active role in inflammation and fibrosis.

### 6.1. Platelets Chemokines Molecules

#### 6.1.1. Platelet Factor 4 (PF4)/CXCL4

In 1969, Niewiarowski and Thomas described one of the most abundant basic proteins stored in platelet α-granules, PF4, or CXC chemokine ligand 4 (CXCL4) [89]. Platelet factor 4, a 70 amino acid heparin-binding protein, was the first member of the chemokine family identified in platelets and is one of the most abundant chemokines contained within platelet α-granules. PF4 is released from activated platelets in a P-selectin-dependent manner and exerts effects on numerous other cells [90,91]. The biosynthesis of PF4 is almost exclusively limited to megakaryocytes from which mature circulating platelets are derived. In platelets, this chemokine accounts for ~2% of the total α-granular volume. A recent study also reported that low levels of PF4 are synthesized in activated human monocytes [92].

PF4 has pleiotropic effects in hemostasis and thrombosis, which include procoagulant and anticoagulant properties; it not only interferes with the action of heparin, thereby increasing coagulation [93], but also has an angiostatic effect because it interferes with the proliferation and chemotactic of endothelial cells. It binds to the platelet surface, which suggests that this molecule also has the ability to regulate cell interactions [13,85,94]. PF4 also a strong chemoattractant for neutrophils, monocytes, and fibroblasts. PF4 can trigger neutrophil activation and adhesion to endothelial cells, phagocytosis in monocytes, and T lymphocyte chemotaxis [90].

PF4 has been shown to be elevated in the DKD. Gruden et al. [95] demonstrated that PF4 values were significantly higher in insulin-dependent diabetic patients with macroalbuminuria than normo-, microalbuminuric diabetic and healthy controls.

#### 6.1.2. Beta-Thromboglobulin (β-TG)

Another important platelet protein is β-TG, which was first described by Moore et al. in 1975 [94]. β-TG is a lysis product of PF4 and platelet base protein (PBP) and it has 50% structural similarity with PF4 [13,85,94]. It constitutes as much as 10% of the content of platelet α-granules and is released because of the influence of platelet activators such as ADP, collagen, immune complexes, and thrombin. β-TG has a plasma half-life of about 100 min and is excreted in the urine [94]. It also has a role as a leukocyte chemoattractant.

As a platelet-specific protein, the concentration of β-TG in plasma increases when platelets are activated [13,85,96], which makes it useful as an indicator of platelet involvement in vascular disease. In patients with chronic renal failure, plasma levels of β-TG and PF4 were significantly increased compared to in normal subjects [97,98]. This increase in β-TG is highly correlated with BUN and creatinine and has a very significant negative correlation with creatinine clearance [97]. In patients with insulin-dependent diabetes with nephropathy, urinary β-TG was found significantly elevated compared to in normal controls. There was a strong correlation between urinary β-TG excretion and both the plasma creatinine concentration and the plasma beta microglobulin concentration, which means that β-TG in urine correlates with glomerular filtration indicators [96].

#### 6.1.3. CCL5 (RANTES)

RANTES (regulated on activation, normal T-cell expressed and secreted, also known as CCL5) is a small protein composed of 68 amino acids and belongs to the chemokine family. RANTES is stored in platelet granules and is released when platelets are activated as a component of microvesicles. RANTES is a chemoattractant for eosinophils, monocytes and T lymphocytes. It induces leukocyte adhesion and migration by binding to specific receptors in the family of seven transmembrane G-protein-coupled receptors (GCPR), namely CCR1, CCR3, CCR4, and CCR5 [90,99].

The release of RANTES in the form of microvesicles causes it to be transported and deposited into injured or activated endothelium. This binding then enhances monocyte recruitment, modulating the occurrence of adhesion and rolling on the endothelium [90].

A significant increase in the expression of RANTES was associated with increased risk of developing T2DM [100]. RANTES has also been detected in renal tissue. Elevated expression of RANTES was shown in biopsy samples of patients with DKD. This expression was shown mainly in tubular epithelial cells of the patients and correlated directly with the magnitude of proteinuria and interstitial cell infiltration. These results suggested that the involvement of RANTES in the pathogenesis of DKD is possible through the recruitment and activation of macrophages/monocytes and lymphocytes [101].

### 6.2. Platelets Growth Factors and Angiogenic Factors

Platelet α-granules contain numerous kinds of growth factors that are involved in cell growth, including epidermal growth factor (EGF), hepatocyte growth factor (HGF), insulin-like growth factor (IGF), and transforming growth factor β (TGF-β). Other angiogenic factors release from α-granules including platelet-derived growth factor (PDGF), vascular endothelium growth factor (VEGF), and fibroblast growth factor (FGF) [102].

#### Platelet-Derived Growth Factor (PDGF)

The platelet-derived growth factor (PDGF) family consists of four isoforms (PDGF-A, PDGF-B, PDGF-C, and PDGF-D) that are secreted as homodimers or heterodimers. Two structurally similar A- and B-polypeptide chains (PDGF-A and PDGF-B) can form two disulfide-bonded homodimers (PDGF-AA and PDGF-BB) and one heterodimer (PDGF-AB), while the isoforms PDGF-C and PDGF-D are released as homodimer (PDGF-CC and PDGF-DD) [55,103,104]. The PDGF signals are mediated by two types of PDGF receptors (PDGFRs), known as PDGFR-α and PDGFR-β. PDGF-A and PDGF-C bind to the α-receptor chain only, whereas PDGF-D binds predominantly to PDGFR-β and PDGF-B binds to both the α- and β receptor chains [103].

PDGF was initially identified as a component of platelets and is stored in the α-granules, from which it is released into the extracellular environment following activation of the platelets by numerous substances, including platelet activating factor, thrombin, collagen, and immune complexes [105]. PDGF is also produced by many other cell types, including renal cells such as glomerular endothelial cells, mesangial cells, tubular epithelial cells (including collecting duct cells), podocytes, and arterial smooth muscle cells. It is also expressed in other cells such as macrophages [104,106].

PDGF may play a role in inflammatory and proliferative glomerular diseases. It has been shown to play a role in mesangial cell proliferation [60,105,107], is involved in the regulation of matrix synthesis [60,105], and is a potent profibrotic factor in the kidney [108]. PDGF also stimulates the release of TGF-β by mesangial cells and is a potent chemoattractant for mesangial cells [109].

Several studies in animal models and humans have suggested that PDGF may be involved in the pathogenesis of DKD. Nakagawa et al. [110] have demonstrated increased expression of PDGF-B and PDGFR-β in glomeruli in streptozotocin (STZ)-induced diabetic rat models compared to in control rats. They also showed the presence of PDGF-B or PDGFR-β in mesangial cells and in visceral epithelial cells. PDGF administration in a hyperglycemic Goto–Kakizaki rat model of non-insulin-dependent diabetes led to an acute increase in mesangial cell proliferation and activation, but no progressive changes in kidney function or structure [107]. In experimental DKD mice models, Suzuki et al. [111] also showed enhanced PDGFR-β signaling that contributed to the progress of DKD in vivo, with increased oxidative stress and mesangial expansion. In human studies, Langham et al. [112] reported a marked increase in both gene and protein expression of PDGF-A and PDGF-B in biopsies from patients with DKD compared with control tissue.

### 6.3. Platelet CD40L (CD154)

CD40L is a cell surface receptor, transmembrane protein that belongs to the tumor necrosis factor-R (TNF-R) family, which structurally related to TNF-α. Activated platelets also express CD40L. Platelets are known as the predominant source of soluble CD40L (sCD40L), which becomes expressed after platelet stimulation with thrombin, collagen, or ADP [113].

Although originally isolated on CD4+ T cells, CD40L has also been identified on other cells of the immune system (lymphocyte, mast cells, basophils, eosinophils, dendritic cells, and natural killer cells). CD40L is not only expressed on immune cells, but also on non-immune cells. Subsequent research showed that CD40L and CD40 are also present on several cells in the vasculature, including endothelial cells, smooth muscle cells, monocytes, and macrophages [114,115].

CD40L is present not only in a membrane-bound form (mCD40L), but also as a soluble molecule (soluble CD40L/sCD40L). Studies have indicated that 95% of the circulating CD40L present in platelets. Platelet CD40L is also functionally active in multiple domains. This suggests that platelet stimulatory events should be considered in the biological and pathological context of CD40L function [113,114].

Expression of CD40L on platelets has been shown to provide a communicative link between innate and adaptive immunity. Activated platelets enhance lymphocyte adhesion to endothelial cells and trigger T cell responses and migration to inflammatory areas. CD40L binding to its receptor on B cells is known to induce B-cell proliferation, promote B-cell differentiation, block B-cell apoptosis, and mediate antibody class switching [113,114].

Platelet CD40L-CD40 may play a central role in the link between thrombosis and inflammation. Interaction between CD40 in vascular cell membranes and platelet CD40L may trigger a variety of various inflammatory responses, including expression of inflammatory adhesion receptors (e.g., E-selectin, VCAM-1, and ICAM-1), and tissue factor (TF), as well as initiating the release of chemokines (e.g., MCP-1) IL-6, and IL-8 [116]. This process enhances platelet–leukocyte adhesion and generates signals for the recruitment and extravasation of leukocytes to sites of injury, which directly initiate an inflammatory response of the vessel wall [116,117]. sCD40L also shows prothrombotic activity by promoting thrombus formation. In addition, direct binding of sCD40L to glycoprotein (GP) IIb/IIIa promotes and stabilizes platelet thrombosis under high shear rates [117]. GP IIb/IIIa is known to be involved in sCD40L production because GP IIb/IIIa antagonists attenuate the release of sCD40L from activated platelets in vitro [118].

Patients with T1DM or T2DM have both been reported to have significantly higher sCD40L levels than controls. Elevated CD40L levels and inducible release from platelets of diabetic patients also have been shown by Nerea Varo et al. [119]. Platelets from diabetic patients contained higher levels of intracellular CD40L than controls and thrombin stimulated greater platelet sCD40L release in diabetic patients compared to controls. Glucose and AGEs also induced platelet sCD40L release and CD40L expression.

A prospective study by Lajer et al. [120] reported on the follow up of 443 T1DM patients with DKD and a control group of 421 patients with longstanding T1DM and persistent normoalbuminuria. Their study showed that plasma sCD40L levels were higher in patients with DKD compared to normoalbuminuric patients. Another study of T1DM patients showed that increased concentrations of soluble CD40 ligand may help to identify who are at risk of developing incipient nephropathy later in life [121].

Podocytes are also known to express CD40L. Activated platelet supernatants and platelet-derived CD154 stimulate MMP-9 secretion by podocytes, suggesting a potential contribution for platelets and platelet CD154 to matrixmetalloprotease-9 (MMP-9) dysregulation in glomerular inflammation. The pathological proteolytic degradation of the GBM involves proteolytic enzymes, such as MMPs, produced by glomerular cells in inflammatory processes. Up-regulation of MMP-9 expression can be found in several glomerular diseases accompanied by proteinuria, including DKD. Increased plasma or urinary MMP-9 precedes albuminuria in diabetic patients and MMP-9 deficiency limits diabetic kidney injury in the mouse, suggesting direct involvement of MMP-9 in the alteration of the permeability of the filtration barrier [122].

### 6.4. Platelet-Adhesion Molecules and Cellular Interaction

Adhesion molecules are a diverse group of ligand/receptor molecules that facilitate intercellular adhesion or adhesion of cells to ECM. In both healthy and diseased conditions, cell adhesion molecules are essential in various processes such as circulation and movement of leukocytes, cell differentiation, and tissue organization for maintenance of tissue architecture, as well as activation of and communication between immune cells [123].

During acute and chronic inflammatory disease conditions, the expression of adhesion molecules is known to be markedly increased in tissues [124]. In T2DM patients, platelets are characterized by increased expression of adhesion molecules. The increase in circulating soluble adhesion molecules in T2DM can be considered as an indication of endothelial damage and leukocyte activation. [124].

Adhesion molecule-mediated leukocyte binding also contributes to kidney diseases, such as tubulointerstitial nephritis, allograft rejection, and renal I/R injury [125]. In DKD, leukocytes (mainly monocytes/macrophages) accumulate in the glomeruli and the interstitium and their infiltration is mediated by adhesion molecules [29].

#### 6.4.1. P-Selectin

P-selectin (CD62P), a well-known marker of platelet activation, is a highly glycosylated membrane glycoprotein that belongs to the adhesion molecule selectin family. P-selectin is deposited in specific granules localized in the α-granules of platelets and in endothelial cells in the membrane of the Weibel–Palade body [13,85]. P-selectin is rapidly redistributed from the α granule membrane to the platelet surface upon platelet stimulation [126].

Under physiologic conditions, selectins are not expressed on renal endothelial cells [125]. Increased expression of P-selectin has been reported in kidney tissue of patients with DKD and other glomerular diseases [88,125,127]. The involvement of P-selectin has also been investigated by Omoto and Hirata. Omoto et al. [126] have reported in their study that CD62P levels were significantly higher in patients with DM than in the normal control group and in patients with nephropathy compared with patients without complications [88]. Hirata et al. [127] have investigated the expression of P-selectin in patients with DKD compared to patients with other glomerular diseases patients (including minimal change nephrotic syndrome, membranous nephropathy, IgA nephropathy, mesangioproliferative glomerulonephritis, and lupus nephritis). They observed significantly increased levels of selectin expression in both the glomeruli and interstitium of kidney tissue of the patients with DKD.

P-selectin is the main mediator of platelet-leukocyte aggregation. It interacts with P-selectin glycoprotein ligand-1 (PSGL-I) on the surface of monocytes and neutrophils that have PSGL-1 ligands appropriate for P-selectin and, thus promotes platelet and endothelial cell adhesion to leukocytes, chemotaxis, leukocyte adhesion, and migration. It also induces activated monocyte differentiation to become macrophages that participate in inflammation [71]. PSGL-1 has high affinity to P-selectin where its interaction increases the avidity and affinity of leukocyte 2 integrin and triggers phosphorylation of ERK-1/2 in neutrophils [128]. The binding of platelet P-selectin to PSGL-1 on neutrophils also appeared to contribute to the amplification of ROS generation by neutrophils, as the ROS generation capacity of neutrophils was significantly increased when neutrophils form hetero-aggregates together with platelets [129]. In addition, P-selectin on the surface of activated platelets induced synthesis of IL-8, MCP-1, and TNF-α by monocytes, as well as expression of tissue factors, when synergized with a platelet-activating factor (PAF) and/or RANTES [53].

P-selectin also has a role in platelet aggregation. In a mouse model, increased levels of soluble P-selectin accelerated hemostasis [114]. P-selectin binding to PSGL-1 is also known to induce platelet activation, which enhances platelet aggregation and thrombus formation [128]. αIIbβ3-integrin (GPIIb/IIIa) activation is triggered by increased calcium spikes in platelets because of the binding of P-selectin to soluble PSGL-1 or sulfatide membranes. This triggers platelet aggregation and the formation of platelet–leukocyte aggregates [128]. According to Merten et al. [130], P-selectin participated in stabilizing the initial αIIbβ3-integrin–fibrinogen interactions, allowing the formation of large and stable platelet aggregates.

#### 6.4.2. Thrombospondin-1 (TSP-1)

TSP-1 is a commonly secreted glycoprotein that belongs to the thrombospondin (TSP) family. This protein was named a thrombin-sensitive protein (TSP) when it was first identified through its release in response to the activation of platelets by thrombin. According to molecular organization, the thrombospondin gene family, which comprises proteins encoded by five separate genes, is divided into two subfamilies, types A and B. TSP-1 and TSP-2 belong to subgroup A and subgroup B consists of TSP-3, TSP-4, and TSP-5 [131]. TSP-1 is expressed by a variety of cell types including platelets, vascular smooth muscle cells, and mesangial cells (MCs) and is frequently expressed at sites of inflammation and wound healing [132].

Thrombospondins are the major secretory protein products of activated platelet α-granules, which bind to the surface of activated platelets upon platelet stimulation. Thrombospondins play an important role in platelet aggregation. On the surface of activated platelets, TSP forms a specific complex with fibrinogen and this interaction is an important step in the aggregation process [133,134].

Thrombospondins are multifunctional glycoproteins that are involved in the regulation of angiogenesis, cell proliferation, cell adhesion and migration, modulating platelet aggregation, apoptosis, activation of protein kinase, and TGF-β activity [132,135]. As a typical matrixcellular protein, TSP-1 is tightly regulated by cytokines such as PDGF, FGF-2, or TGF-β, and by various possible interactions with other cytokines, receptors, and proteases [132]. TSP-1 has been shown to be a crucial activator of latent TGF-β, a mediator of angiostatic signals, an inhibitor of inflammation through its CD47 interaction, and a modulator of matrix metabolism by direct or indirect matrix metalloproteinase inhibition [136].

TSP-1 was found to be an important regulator of pathophysiological changes during renal disease in various animal models of renal disease and in patients with DKD. Daniel et al. [137] investigated the role of TGF-β in type 1 diabetes in TSP-1-deficient mice. The concentration of active TGF-β within glomeruli was found to be significantly lower in TSP-1-deficient mice than in wild-type mice. The development of DKD was attenuated in TSP-1-deficient mice as demonstrated by a significant reduction in glomerulosclerosis, glomerular matrix accumulation, podocytes injury (damage), glomerular hypertrophy, renal infiltration with inflammatory cells (macrophages and T lymphocytes), and renal functional parameters. The authors concluded that TSP-1 is an important activator of TGF-β in DKD in vivo.

A study of TSP-1 in patients with DKD also showed that serum TSP-1 is significantly higher in patients with DKD than in normal control subjects [132,135]. TSP-1 levels correlate with the degree of renal impairment and vascular disease in patients. These aspects were related to the ability of TSP-1 to enhance latent TGF-β bioactivity, which is a profibrotic cytokine that mediates glomerular response to injury through glomerular hypertrophy, matrix expansion, and glomerulosclerosis. The functional activity of the TSP-1/TGF-β axis was shown to be involved in the tubulointerstitial injury of patients with DKD [135].

### 6.5. Platelet-Leukocyte Interaction

Under inflammatory conditions, platelets greatly affect leukocyte recruitment to areas of inflammation in many tissues, although platelets are mostly located intravascularly. Activated platelets interact with leukocytes during all steps of the extravasation cascade and coordinate their recruitment to sites of inflammation and infection [138]. Platelets in the vasculature maintain their ability to bind and recruit leukocytes and aid their extravasation by secreting cytokines and chemokines (CCL5 and CXCL4). Deposition on the surface of endothelial cells at the inflammation areas promote the intravascular rolling and extravasation of leucocytes and by enhancing adhesion, primarily through P-selectin–PSGL1 interactions [139,140].

**Figure 1:**
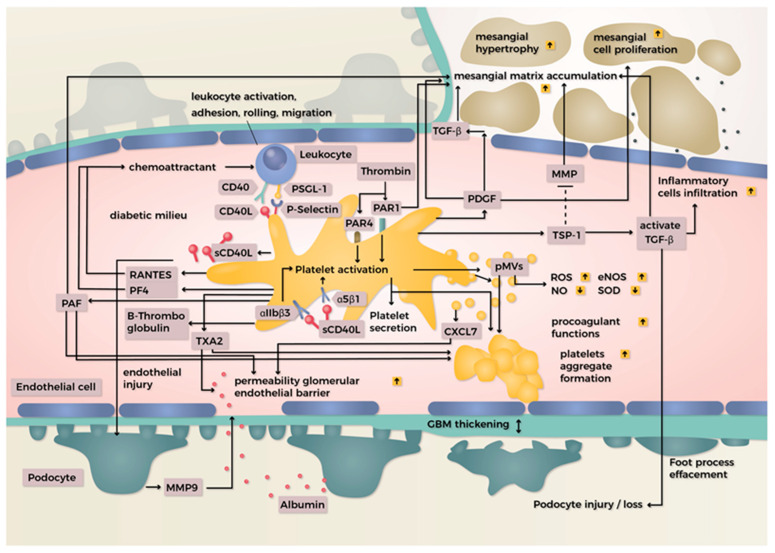
Mechanisms of platelet involvement in the pathogenesis of diabetic kidney disease. Under diabetic conditions, platelet hyperreactivity is characterized by increased activation and signaling of platelet receptors, leading to platelet adhesion, activating other platelets to form aggregates, and contributing to the coagulation cascade. Activated platelets directly bind to leukocytes through a P-selectin–PSGL-1, CD40-CD40L interaction and stimulate leukocytes extravasation. Soluble CD40L produced by activated platelet binds to αIIbβ3 and α5β1 integrin and mediates platelet activation. sCD40L may affect podocytes, leading to an increase in the expression of MMP9 and enhancing glomerular permeability. PAR receptors are also expressed in platelets, which can be activated by thrombin signaling, thus mediating platelet activation. Platelet activation releases cytokines and chemokines such as RANTES/CCL5, PF4/CXCL4, and β-TG/CXCL7. Platelets contain numerous growth factors such as PDGF, which contribute to mesangial cell proliferation and matrix accumulation in DKD. With TSP-1, PDGF also stimulates and activates the release of TGF-β, responsible for mesangial matrix accumulation, renal infiltration with inflammatory cells, and podocyte damage. TXA2 and PAF secrete from platelets, increase platelet aggregation, and enhance glomerular permeability, inducing proteinuria. PAF also play a part in subsequent inflammatory reaction and stimulate ECM deposition. Platelets also release abundant MVs and induce the production of ROS, decrease NO, and inhibit the activities of eNOS and SOD. Platelet MVs also contribute to the injury of glomerular endothelial cells by releasing CXCL7.

Platelets interact with immune cells through a receptor–ligand interaction, thus enabling the presence of cell–cell communication. In addition, platelets contribute to the innate immunity response by interacting with circulating neutrophils or monocytes. Platelet–neutrophil and platelet–monocyte interactions are mediated by the platelet P-selectin/ligand PSGL-1 on the neutrophil and monocytes axis, causing migration and inflammatory responses (Figure 1). The binding of platelet P-selectin to PSGL-1 on neutrophils contributes to the amplification of ROS generation by neutrophils, which contributes to pathogen killing and other pathophysiological effects [75,129].

A recently described effect of platelet–leukocyte interaction is neutrophil extracellular traps (NETs) formation. Platelets can induce NETs formation via several pathways. TLR4-mediated interaction between platelets and neutrophils can result in NETs formation, especially in systemic inflammation [75,129,140,141,142]. The binding of platelet integrin aIIbβ3 to neutrophil CD11b via fibrinogen together with stimulation of the chemokine-neutrophils by CXCL4/CCL5 has been identified as another mechanism for NETs formation during sterile inflammation [129]. Platelet inhibition by clopidogrel resulted in lower numbers of intrarenal NETs, leading to enhanced tissue damage and preserved renal function in murine acute kidney injury (AKI) [143].

NETs were the first type of extracellular traps (ETs) to be discovered. NETs formation has been linked to the progression of several kidney diseases [144]. Diabetes mellitus and its complications are also associated with increased NETs release [145,146]. However, the involvement of NETs in DKD is currently unclear. Other cells such as macrophages can also produce ETs, known as macrophages extracellular traps (METs). Besides ample evidence that platelets modulate NETs, there is also evidence that they modulate the formation of METs [147]. In a mouse model of rhabdomyolysis-induced kidney injury, heme-activated platelets enhanced the production of METs through increasing intracellular generation of ROS and histone citrullination [148]. Since monocytes and macrophages are key players in the pathogenesis of diabetic nephropathy, involvement of METs in this disease is plausible. So far, no reports have described the role of METs in DKD.

### 6.6. Platelet Derived Microvesicles

Microvesicles (MVs), formerly known as “microparticles” (MPs), are a type of extracellular vesicle. Platelet-derived MVs are produced by platelet activation or by physical stimulation under various conditions. MVs are spherical-like structures of 0.1 to 1.0 µm in diameter and are encapsulated by a lipid bilayer that typically possesses membrane and cytoplasmic contents (including lipids, proteins, mRNA and/or miRNA, and DNA) of their parent cells [149]. MVs are released during apoptosis and cellular activation of many cell types. Under physiologic circumstances, MVs in the peripheral blood are derived mostly from platelets and endothelial cells, whereas in pathological contexts (i.e., under influence of cytokines, thrombin, endotoxins or physical stimuli, as well as shear stress or hypoxia), numerous kinds of cells have been observed to release MVs, including erythrocytes, monocytes, lymphocytes, vascular smooth muscle cells, and many other cell types [149,150].

Microvesicles from different cellular sources have been implicated both in animal models of DKD [151] and human DKD samples [126,150,152]. Yu et al. [153] found that DKD patients have higher levels of MVs than healthy subjects, which may be due to numerous factors, such as hyperglycemia, dyslipidemia, and oxidative stress [26]. Uil and Lu et al. [152,154] also showed that diabetic patients with nephropathy had elevated numbers of plasma platelets MVs and endothelial MVs compared to healthy controls. Furthermore, the study by Rodrigues et al. [150] observed that T2DM patients with nephropathy presented higher levels of MVs derived from platelets (PMVs), leukocytes, endothelial cells, and tissue factor, which are influenced by gender, glycemic control, and 25 (OH)D levels, suggesting that T2DM patients with DKD presented higher circulating MVs levels that correlated with metabolic alterations.

In a rat model of STZ-induced diabetes, Zhang et al. [151] demonstrated the effects of PMV on glomerular endothelial injury in early DKD. Their study showed that the increased levels of PMV induced the production of ROS, decreased NO, inhibited the activities of endothelial nitric oxide synthase and superoxide dismutase (SOD), increased the permeability of the glomerular endothelium barrier, and reduced the thickness of endothelial surface layer. They also demonstrated that PMVs contribute to injury of glomerular endothelial cells by releasing CXCL7 (Figure 1).

### 6.7. Thromboxane A2

TXA2 is produced by the sequential enzymatic metabolism of arachidonic acid through the cyclooxygenase or prostaglandin H (PGH) synthase pathway. Under physiological conditions, TXA2 is rapidly hydrolyzed (the half-life of TXA2 is 32 s) to TXB2, which is stable but physiologically inactive. Thus, the effects of TXA2 are largely confined to tissues near the source of its production. Due to its stability, measurement of TXB2 has been a useful marker for assessing the production of TXA2 in vivo [155,156].

TXA2 is a biologically active derivative of arachidonic acid. Thromboxane receptors (TP receptors) have been shown to be involved in the activation of blood platelets. Its activity is mediated by binding to TP-specific G protein-associated receptors. It is now clear that TP receptors also exhibit a wide distribution across different cell types and among different organ systems. TP receptors are known to play a role in normal renal function, and evidence suggests that TP receptors may mediate renal damage in disease states [156].

The level of TXA2 from platelets increases in T2DM and inhibition of platelet aggregation by a TXA2 synthase inhibitor slows down progression of nephropathy. Studies by Okumura et al. [155] in rat models of T2DM showed that the progress of nephropathy (increase in proteinuria) paralleled an increase in the urinary excretion of TXB2. Glomerulosclerosis and thrombi in the glomerular capillaries were seen in the untreated diabetic rats. Diabetic rats with an inhibitor of TXA2 synthase had less formation of glomerular thrombi and proteinuria was suppressed. In addition, it was shown that TXA2 affected the glomerular filtration size selectivity, as well as the GFR. TXA2 also plays a role in regulating gene expression in the glomerular matrix, inducing mRNA transcription for type IV collagen, laminin, and fibronectin, while suppressing heparan sulfate proteoglycan synthesis in DKD [155].

### 6.8. Platelet Activating Factor (PAF)

PAF is a potent phospholipid autacoid (1-0-alkyl-2-acetyl-sn-glycero-3-phosphocoline), a lipid mediator with important pro-inflammatory effects that was initially isolated from stimulated basophils. PAF is a bioactive phospholipid produced by phospholipase A2 and acetyl-transferase, which acts on the cell membrane glycerolipid choline. PAF is mainly produced by platelets, but also by other cells such as neutrophils, mast cells, endothelial cells, macrophages, and glomerular mesangial cells [71,157]. PAF released from platelets may thus trigger platelet aggregation, activation, and degranulation, a basic mechanism for the formation of the platelet plug during hemostasis [157,158].

PAF has a multitude of biological effects pertinent to glomerular injury. During kidney inflammation, PAF is synthesized by inflammatory cells that infiltrate the tissue and/or by mesangial cells. PAF acts on a G-protein-coupled receptor (PAFR) expressed in monocytes/macrophages, polymorphonuclear leukocytes, platelets, endothelial cells, and many other cell types. PAF modulates the subsequent inflammatory reaction. These include chemotaxis and activation of leucocytes, complement activation, and contraction and stimulation of mesangial cells to produce eicosanoids and ROS [157,159]. PAF has a biological effect when it binds to its receptor. It stimulates B lymphocytes to produce IgG and IgE, and to induce macrophages to secrete IL-1, IL-6, and TNF [71].

Increased levels of PAF were found to be present under basal conditions in the blood of microalbuminuric insulin-dependent diabetic (IDD) patients, but not in normoalbuminuric IDD patients and in controls [160]. Chignard et al. [158] also compared urinary PAF excretion in subjects who were diagnosed with non-insulin-dependent diabetes mellitus (NIDDM) with healthy controls They found that the NIDDM subjects produced significantly higher levels of urinary PAF and urinary PAF excretion was significantly correlated with microalbumin excretion.

The enhanced glomerular permeability induced by PAF has been shown to lead to significant proteinuria in experimental animals. Perico et al. [161] investigated the effect of platelet-activating factor (PAF) on glomerular permeability to macromolecules in rats. PAF infusion induced a progressive and significant increase in protein excretion, and this was completely reversible after infusion was stopped. This suggests that PAF causes proteinuria by altering the glomerular size-selective properties (Figure 1). As a result, the transmural passage of large dextran molecules is selectively increased. This change was also dependent on the calcium concentration in the extracellular medium.

In the model of obstructive nephropathy, PAFR signaling contributes to a pro-inflammatory environment that favors the fibrotic process by collagen deposition and regulation of ECM. It may promote epithelial-to-mesenchymal transition (EMT), which has recently been shown to generate renal dysfunction and progressive organ failure [159]. In a DKD model, PAF stimulated ECM deposition in human mesangial cells via activation of the PKC-TGF-β1 axis (Figure 1). Stimulated by high glucose and lysophosphatidylcholine (LPC), it also induced fibronectin (FN) and collagen IV secretion. The accumulation of ECM deposition increases the risk of glomerular fibrosis [162].

### 6.9. Protease-Activated Receptors (PARs)

Protease-activated receptors (PARs) are members of the family of transmembrane G protein-coupled receptors (GPCRs). Four members of the PAR family (PAR1-PAR4) have been identified. They are activated by endogenous serine proteases, such as thrombin (acting on PAR 1, 3, and 4) and trypsin (acting on PAR2). Members of the PAR family are expressed in various cell types including immune cells, platelets, smooth muscle cells, and endothelial cells. PAR1 and PAR4 are detectable in human platelets and, through cleavage PARs, thrombin activates platelets (Figure 1) [163,164].

Kahn et al. [164] demonstrated that PAR1 and PAR4 are functionally expressed in human platelets and that these receptors are responsible for thrombin signaling in platelets. They also reported that PAR1 mediates the platelet response even at low thrombin concentrations and that it is required for rapid and vigorous platelet response. In contrast to PAR1, PAR4 mediates platelet activation only at high thrombin concentrations and PAR4 signaling does not appear to be required for platelet activation when the PAR1 function is intact.

PAR-1 and PAR-4 have been shown to contribute to mechanisms in several kidney injury models, including AKI and DKD patients [165]. PAR-1-mediated platelet activation was demonstrated in diabetic patients with coronary artery disease (CAD) and expression increased compared with nondiabetic CAD patients [166].

Gianella et al. [167] demonstrated for the first time that PAR-4 as a mediator of platelet activation promoted the release of activated PMVs through a calcium calpain-dependent mechanism and the expression of PAR-4 is upregulated by chronic hyperglycemia in poorly controlled (HbA1c > 7.0%) T2DM patients. PAR-4 also had a pro-inflammatory effect on the release of IL-6 from macrophages treated with platelets from poorly controlled patients.

## 7. Therapeutic Perspectives of Platelet Inhibitors in DKD

In the clinical treatment of DKD, it is common to seek methods and drugs that can effectively prevent diabetic microangiopathy. In particular, the control of microalbuminuria is an important readout for the treatment of this disease [168]. The application of antiplatelets drugs has been assessed in experimental (Table 3) or clinical study of DKD (Table 4).

### 7.1. P2Y12 Receptor Antagonists

#### 7.1.1. Clopidogrel

Clopidogrel is a potent and specific inhibitor of the ADP P2Y12 receptor, causing a decrease in platelet activation and aggregation. Structurally, clopidogrel belongs to the thienopyridine group of antiplatelet drugs and specifically inhibits ADP-dependent platelet aggregation and adhesion via inhibition of purinergic P2Y12 receptors [169]. Clopidogrel may act as an anti-inflammatory and anti-thrombotic. Clopidogrel withdrawal in patients with diabetes on long-term (12 months) dual antiplatelet therapy (aspirin plus clopidogrel) showed a significant increase in effects on platelet aggregation (ADP aggregation) and inflammatory biomarkers (C-reactive protein, P-selectin expression in resting platelets, and P-selectin expression in ADP-stimulated platelets) [197]. Clopidogrel reduces the incidence of thrombosis in PF4/H antibody-positive patients by inhibiting platelet activation [198]. Clopidogrel also has anti-fibrotic properties via the TGF-β pathway [169].

The benefits of clopidogrel in DM has been extensively studied in patients and animal models. Zheng et al. [169] demonstrated clopidogrel-induced platelet inhibition in the improvement of diabetes-induced renal fibrosis. Their study showed that in diabetes-induced mice, renal outcome was significantly improved, indicating that fibrosis, when accompanied by accumulation of FN, could be substantially reduced by clopidogrel treatment. They also reported a decrease in glucose levels in the last month of a 3-month clopidogrel treatment. Their results suggest that activation of TGF-β, CTGF, and MAP kinase is an early profibrotic signaling event, resulting in significant FN accumulation at an early time point and a return to baseline at a later time point.

In contrast to the results of animal study, the therapeutic effect of clopidogrel in DM patients showed a weak effect in lowering the risk of cardiovascular events. The first systematic review and meta-analysis of randomized controlled trials studies on the efficacy of clopidogrel for the treatment of acute coronary syndromes or ischemic stroke in patients with or without DM was published by Liang et al. in 2020. The study comprising more than 40,000 participants (with and without DM) revealed that the combination of clopidogrel and aspirin significantly reduced the risk of any cardiovascular event in both patients with and without DM who had ischemic cardiovascular disease. The effect of clopidogrel and aspirin in individuals with diabetes appeared to be smaller than in those without diabetes, although this difference did not achieve statistical significance [199].

The CHARISMA study involving more than 15,000 patients with DKD showed harmful effects of clopidogrel. This study was performed by Dasgupta et al. [182] in 2009. It examined the risks and benefits of long-term clopidogrel administration (75 mg/day) in patients with DKD. Patients with nephropathy randomly assigned to clopidogrel had significantly increased cardiovascular (CV) mortality and overall mortality, whereas symptomatic diabetic patients without nephropathy who received clopidogrel showed significant reductions in the combined primary end point of CV death, myocardium infarct, and stroke. However, there was no significant difference in CV mortality or overall mortality compared to placebo (aspirin administration). These results suggest a beneficial effect of clopidogrel use in diabetic patients without nephropathy, but no benefit in patients with DKD.

#### 7.1.2. Ticagrelor

Ticagrelor is a noncompetitive, reversible, direct-acting P2Y12-receptor antagonist with a strong platelet-inhibition effect. Ticagrelor binds reversibly to inhibit receptor signaling and subsequent platelet activation and does not require metabolism for activity. In contrast, clopidogrel and prasugrel are irreversible inhibitors of the P2Y12 receptor. Importantly, ticagrelor treatment reduced the incidence of cardiovascular mortality, myocardial infarction, and stroke [200,201].

In addition to its platelet-inhibition effect, ticagrelor has been shown to be effective in the treatment of acute coronary syndromes (ACS) and kidney disease. The effect of ticagrelor in DKD was examined by Uil et al. [179]. They studied the effect of platelet inhibition by ticagrelor in an STZ-induced T1DM mouse model. Ticagrelor treatment in diabetic mice lowered urinary albumin excretion and reduced mesangial matrix expansion, podocyte effacement, and glomerular endothelial cell injury, which includes loss of endothelial fenestrations, ICAM-1 expression, and PECAM expression. Ticagrelor treatment also prevented collagen IV deposition and macrophage infiltration in the tubulointerstitium, leading to decreased tubulointerstitial fibrosis, inflammation, and tubular apoptosis. This study showed that ticagrelor is a promising treatment for reducing the development or progression of DKD.

Platelet reactivity is higher in diabetic patients than in healthy controls. Platelet hyperreactivity plays a role in the increased thrombotic risk of diabetic patients, and DM is clearly a risk factor for high residual platelet reactivity (HRPR). In one of the largest cohort studies by Nardin et al. [202], comprising 224 patients with a recent ACS treated by ticagrelor, 38.4% (86) of the patients were diabetic. Researchers reported that DM is independently associated with higher platelet reactivity and found a significant linear association between HRPR and glycosylated hemoglobin levels, but not with fasting glycaemia. Moreover, according to a recent meta-analysis by Alexopoulos et al. [203] that included 445 patients from eight studies who had platelet reactivity (PR) assessment when treated with a maintenance dose of ticagrelor, DM was associated with a low probability for low PR, suggesting an association between diabetic status and PR during ticagrelor maintenance therapy. In a later study, they observed 777 patients with ACS who underwent percutaneous coronary intervention treated with prasugrel or ticagrelor. In contrast to the previous study, they found that patients treated with ticagrelor had a lower overall PR than patients on prasugrel regardless of DM status or insulin treatment [204].

Ticagrelor has the potential to enhance the treatment of patients with ACS. A PLATO trial that enrolled 18,624 ACS patients, reported that 4662 (25%) had pre-existing DM. The members of this subgroup more often had multiple CV risk factors, the majority (96%) of which were in T2DM patients. In a sub-study involving 1036 diabetic patients, ticagrelor compared with clopidogrel reduced the number of ischemic events in ACS patients irrespective of diabetic status and glycemic control. Ticagrelor decreased the primary composite outcome of CV death, MI, or stroke and similarly reduced the primary end point, all-cause mortality, as well as stent thrombosis, without any significant increase in overall major bleeding [205].

### 7.2. Serotonin Receptor Antagonist

#### Sarpogrelate

Sarpogrelate is a selective 5-HT_2_A (5-hydroxytryptamine; 5-HT) receptor antagonist. It is an anti-platelet drug that has been used to treat thrombotic diseases and is used clinically for treatment of vascular inflammation and atherosclerosis [170]. Serotonin (5-hydroxytryptamine; 5-HT), mainly released from activated platelets, is a decarboxylation derivative product of the amino acid tryptophan [185]. High levels of plasma serotonin are found in diabetic patients and it is thought to play a role in the mechanisms of diabetic complication [206,207]. Sarpogrelate has been used in patients with DM as an antiplatelet drug alternative for aspirin. Sarpogrelate has been shown to prevent macrovascular [208] and also microvascular complications [183] in patients with T2DM.

Studies have also reported that sarpogrelate can reduce albuminuria in DM patients with early stage DKD [184,185]. In patients with T2DM, Yoo et al. [183] showed that metformin-based antidiabetic therapy with sarpogrelate may reduce the incidence of DKD and slow the progression of nephropathy T2DM. The use of sarpogrelate, an antiplatelet drug, can have a beneficial effect on the progression of renal complications in DM.

A recent animal study suggested a new mechanism by which sarpogrelate acts in DKD. In an animal model of proximal tubular inflammation and fibrosis with hyperglycemia, sarpogrelate was found to be beneficial in diabetic kidney disease by inhibiting macrophage activities and by anti-inflammatory activity [170]. Kim et al. [171] showed that sarpogrelate and cilostazole were effective in ameliorating nephropathic progression in diabetic hypertensive rats. Sarpogrelate reduced albuminuria, collagen deposition, and histopathological changes (renal cortex degeneration, increased glomerular diameter, mesangial expansion, and tubular vacuolation). Moreover, they showed that the combination of cilostazol with telmisartan was additively effective in decreasing urine albumin-to-creatinine ratio (UACR), tissue collagen deposition, and histopathological alterations in the kidney. In nephropathic animals, they also analyzed urinary miRNAs (which can reflect chronic disease-induced nephropathy and therapeutic effects of treatments), whose expressions were repressed by both sarpogrelate and cilostazole. They found that miR-199a-3p is associated with TGF-β1-induced renal fibrosis by targeting CD151.

### 7.3. Phosphodiesterase Inhibitors

#### Cilostazol

Cilostazol (6-[4-(1cyclohexyl-1H-tetrazol-5-yl) butoxy]-3, 4-dihydro-2(1H) quinolinone) is a potent, specific phosphodiesterase 3 (PDE 3) inhibitor with major effects in the prevention of platelet aggregation and vasodilatory, and antiproliferative effects that result from increasing the cellular concentration of cAMP in platelets and vascular smooth muscle. Cilostazol has been shown to have anti-inflammatory, antioxidant, and antibrophilic effects. Due to its antiplatelet and vasodilative properties, cilostazol has been widely prescribed in clinical settings (e.g., for treating peripheral artery disease) and has been shown to be effective in treating coronary artery disease, cerebrovascular disease, and kidney disease [173,174,209]. Cilostazol plays an important role in the prevention and treatment of diabetes complications through a variety of mechanisms, including anti-inflammatory effects, inhibition of vascular smooth muscle proliferation, nerve cells protection, and blood lipid regulation [168]. Cilostazol significantly suppresses spontaneous microaggregation of platelets and platelet activation in patients with T2DM who have an inadequate platelet response to aspirin [172]

The renoprotective effects of cilostazol in diabetic kidney disease were demonstrated in experimental animal studies and in patients with DKD. Park et al. [173] investigated the potential beneficial effects of cilostazol on metabolic disorder-induced renal dysfunctions. They conducted a study in C57BL/6 mice that received a high-fat diet for 22 weeks and were given a single low dose of STZ that led to renal injury. The mice were subsequently treated with cilostazol for 13 weeks. Cilostazol significantly improved UACR and urinary cystatin C levels. It also attenuated the histopathological changes, including glomerular mesangial expansion, tubular vacuolization, apoptosis, lipid accumulation, and tubulointerstitial fibrosis.

In STZ-induced diabetic rats, Wang et al. [172] demonstrated that the renoprotective effects of cilostazol may be mediated by its anti-inflammatory effects. These include inhibition of NF-κB activation, reduced inflammatory cells infiltration, reduced expression of adhesion molecules (VCAM-1 and ICAM-1) mRNA, and inhibition of other pro-inflammatory factors, such as MCP-1 and VEGF expression in the kidneys of diabetic rats.

Cilostazol treatment in STZ-induced type 1 DKD rat models also showed reduced progression of DKD by suppressing renal oxidative stress and improving dyslipidemia conditions [174,175,209]. Cilostazol restored the anti-oxidative capacity by elevating the circulating plasma sRAGE [209], reduced the MDA level, and restored the catalase and GSH levels [174]. Cilostazol also downregulated the expression of NF-κB and TGF-β, which are involved in the progression of renal diseases and thus ameliorate the onset of DKD [174,175,209].

In a 52-week randomized, single-blinded trial in 90 patients with DKD, treatment with oral cilostazol significantly reduced the urinary microalbuminuria and UACR levels. This effect was mediated by an improvement of adhesion molecules (including MCP-1, E-selectin, and sVCAM-1). However, there were no significant changes in body mass index, blood pressure, HbA1c, fasting glucose, lipid profiles, blood urine nitrogen, creatinine, uric acid, and aspartate aminotransferase [186]. Similarly, Jiao et al. [168] investigated 60 T2DM patients with early nephropathy and reported a significant decrease in sICAM-1, MCP-1, and urinary albumin excretion after treatment with cilostazol for 6 months. However, no significant changes in blood pressure, liver and kidney function, or HbA1c were observed after treatment with cilostazol. Another study demonstrated the role of modulation of prostaglandin metabolism in glomeruli, decreased thromboxane, and alteration in the prostacyclin balance, resulting in reduced urinary microalbuminuria [187].

A literature review conducted by Asal et al. [210] in 2017 using the PubMed and Embase databases on the use of cilostazol in diabetes mellitus concluded that cilostazol may be beneficial in patients with T2DM and DKD. However, they suggested that larger studies are needed to assess the benefits and risks of using cilostazol as an alternative agent in treating patients with microvascular complications of diabetes.

### 7.4. Adenosine Reuptake Inhibitors

#### 7.4.1. Dipyridamole

Dipyridamole [DP; 2,6-bis(diethanolamino)-4,8-dipiperidinopyrimido-(5,4-d)-pyrimidine], is a synthetic derivative of pyrimido-pyrimidine. It is an anti-platelet agent that is conventionally used for secondary prevention of transient ischemic attack [211]. In rat models, researchers investigating the effect of dipyridamole showed that in low doses it has a significant preventive effect on diabetes mellitus-induced vascular endothelial dysfunction and nephropathy [211] and also corrected early changes in the diabetic kidney, although blood and urinary glucose levels were unchanged [177]. In STZ-induced diabetic rats, the preventive effect of dipyridamole on the progression of DKD was also demonstrated by a reduction in adenosine levels, which resulted in mild glomerular effects and vacuolation of tubular epithelium. Dipyridamole reduced inflammation by restoring the normal balance between pro- and anti-inflammatory cytokines (IL-1β, IL-10, IL-18, and TNF-α). This study was conducted in vivo and in vitro in human mesangial cells cultured in high levels of glucose and showed reductions in the adhesion molecule, ICAM-1, and in intrinsic apoptosis (caspases-3/8/9) [178].

In line with studies in animal models, Khajehdehi [195] conducted a study in 76 patients with type 2 DKD who received dipyridamole, either alone or in combination with aspirin. These treatments reduced the incidence of proteinuria, with the most prominent effect seen with a combination of drugs. Another study also showed a reduction in proteinuria in patients with DKD using a combination of aspirin and a low dose of dipyridamole (990 mg/225 mg) for 6 weeks, although diabetic control and blood pressure remained unchanged throughout the study [196].

#### 7.4.2. Dilazep Dihydrochloride

Dilazep dihydrochloride is a potent adenosine uptake inhibitor that exhibits antiplatelet, antianginal, and vasodilator properties. In clinical practice, it is generally used as an antithrombotic drug. Several studies have investigated the effects of dilazep dihydrochloride on the development and progression of DKD. In Otsuka Long-Evans Tokushima fatty (OLETF) rats, a T2DM animal model, dilazep administration slowed the increase in urinary protein excretions and reduced the activity of N-acetyl-beta-D-glucosaminidase (NAG) [176]. NAG is a lysosomal enzyme that is present in proximal tubular cells and is an indicator of renal damage [212]. Dilazep hydrochloride prevented glomerulosclerosis and tubular atrophy and reduced positive staining for type IV collagen in the glomeruli of diabetic rats [176]. Dilazep dihydrochloride also prevented anionic charges on the GBM and decreased the urinary excretion of proteins in STZ-induced diabetic rats [213].

In human studies, dilazep dihydrochloride administered to patients with early stage DKD was reported to be potentially useful for improving albuminuria and preventing renal dysfunction in patients with microalbuminuric DKD [193]. Nakamura et al. [194] reported that dilazep dihydrochloride treatment reduced microalbuminuria and the number of urinary podocytes, and that dilazep dihydrochloride may be useful in preventing renal deterioration in podocyte injury that may occur early in patients with DKD. These studies suggest that dilazep dihydrochloride is a valuable new drug for the treatment of diabetic patients with nephropathy.

### 7.5. Prostaglandin PGI2 Analog

#### Beraprost Sodium

Beraprost sodium (BPS) is a novel stable analogue of prostaglandin I2 (PGI2) that can be administrated orally. It has antiplatelet, vasodilation, and antioxidant activities and is cytoprotective [192,214,215]. It inhibits platelet aggregation mediated by cAMP [214,216,217] and is a potent inhibitor of leukocyte chemotaxis by elevating the intracellular CAMP level and inhibiting the influx of extracellular Ca^2+^ in polymorphonuclear leucocytes [218]. BPS has also been reported to improve renal function in animal models and also in humans [180,181,217].

Several studies have evaluated the effect of BPS in DKD animal models and in patients with DKD. BPS improved renal function and protected the kidneys of DKD rats by reducing oxidative stress and inflammatory cytokines, and it decreased the urinary protein excretion of rats with DKD [180,181]. BPS reduced blood glucose, urine output, high-sensitivity C-reactive protein (hs-CRP) [180], urinary protein, creatinine [180,181], IL-6 levels [180], ICAM-1, and macrophages infiltration [181]. It also ameliorated glomerular hyperfiltration by modulating ecNOS expression in afferent arterioles and glomeruli.

The renoprotective mechanism of BPS in patients with DKD has been explored in several studies. BPS treatment for patients with DKD significantly decreased microalbuminuria [189]. BPS was also found to be effective and safe in the treatment of DKD in elderly patients. Jing Xia et al. [190] investigated 100 elderly patients and included additional BPS in their routine treatment for 15 days. The results showed that urinary protein, blood urea nitrogen (BUN) serum creatinine (sCr), and cystatin C were all lower than in the control group. In 26 Japanese patients with DKD and arteriosclerosis obliterans who were followed for 1 year, BPS combined with RAS inhibition prevented the progression of DKD [191]. In a study of 102 patients with type 2 DKD, BPS combined with alprostadil more effectively improved hemodynamics, coagulation function, and renal function, and inhibited expression of RAS-related factors and TNF-α [192].

## 8. Conclusions

Under normal physiological conditions, platelets are in an inactive state. They play a central role in normal hemostasis and coagulation. However, the diabetic milieu causes platelet hyperreactivity. Increased platelet activation orchestrates a broad variety of platelet responses, including secretion of pro-inflammatory factors, platelet–leukocyte interaction and pro-fibrotic responses, which in concert worsen renal function. The application of antiplatelet drugs, such as P2Y12 receptor antagonists (ticagrelor), serotonin receptor antagonists (sarpogrelate), prostaglandin PGI2 analogues (beraprost), and adenosine reuptake inhibitors (dipyrydamole and dilazep hydrochloride) in various animal studies and in T1DM and T2DM patients, has delivered promising results, improving renal function and reducing the progression of DKD disease. Further studies are still needed, especially large-scale randomized studies with long-term follow-up to objectively evaluate their efficacy.

## Figures and Tables

**Table 1:** Classification of DKD 2014 (From Haneda, et al. [24]).

Stage	Urinary Albumin (mg/gCr) or Urinary Protein (g/gCr)	GFR (eGFR) (mL/min/1.73 m^2^)
Stage 1 (prenephropathy)	Normoalbuminuria (<30)	≥30
Stage 2 (incipient nephropathy)	Microalbuminuria (30–299)	≥30
Stage 3 (overt nephropathy)	Macroalbuminuria (≥300) or persistent proteinuria (≥0.5)	≥30
Stage 4 (kidney failure)	Any albuminuria/proteinuria status	<30
Stage 5 (dialysis therapy)	Any status on continued dialysis therapy	

**Table 2:** Inflammatory and immune mediators implicated in DKD.

Immune and Inflammatory Mediator	
Pro-inflammatory cytokines	TNF-α, IL-1, IL-6, IL-10, IL-18
Chemokines molecules	MCP-1 (CCL2), CSF-1
Adhesion molecules	ICAM-1, VCAM-1, P-selectin, E-selectin
Adipokines molecules	ADIPOQ, leptin, resistin

Abbreviation: TNF: tumor necrosis factor; IL: interleukin; PAI-1: plasminogen activator inhibitor-1; MCP-1: monocyte-chemoattractant protein-1; CAM: cell adhesion molecule; ICAM: intercellular CAM; VCAM: vascular endothelial CAM; ADIPOQ: adiponectin.

**Table 3:** Antiplatelets drugs therapy in animal models of DKD.

Therapeutic Intervention	Target of Action	Type of DKD-Model	Species	Treatment Dose and Duration	Renal Effects	Ref.
Clopidogrel	Inhibits the ADP-dependent aggregation and adhesion via inhibition of the purinergic P2Y12 receptor	Streptozotocin (STZ)-induced type 1 diabetes mouse model	Mouse (C57BL/6J)	20 mg/kg b.w./day clopidogrel, for 3 months	↓ Glomerular and kidney hypertrophy, ↓ collagen, fibronectin accumulation and kidney fibrosis	[169]
Sarpogrelate hydrochloride (SH)	5-hydroxytryptamine [5-HT_2_A] receptor antagonist	DKD in a T2DM mouse model (db/m and db/db mice)	Mouse (C57BLKs/J)	SH (30 mg/kg/day) via oral gavage for 12 weeks	↓ Albuminuria and recovered renal structures (mesangial cell expansion and glomerular hypertrophy, increased GBM thickness and podocyte effacement), ↑ serum adiponectin level, inhibited macrophage infiltration, ↓inflammatory mediators (TNF-α and NOS2)	[170]
Sarpogrelate hydrochloride Cilostazole	SH: 5-hydroxy-tryptamine [5-HT_2_A] receptor antagonist Cilostazole: Phosphodiesterase type III (PDE3) inhibitor	Rat model of hypertension/diabetes-induced nephropathy	Spontaneously hypertensive rats (SHRs)	Sarpogrelate (40 mg/kg) or cilostazol (20 mg/kg) via oral gavage 1 week prior to STZ treatment, and the treatment was continued for 4 weeks after STZ	SH: ↓ albuminuria, collagen deposition, and histopathological changes (renal cortex degeneration, increased glomerular diameter, mesangial expansion, and tubular vacuolation). Cilostazol: ↓ UACR levels and collagen deposition in the cortex Both drugs did not affect blood glucose levels or blood pressure, but ↓ urinary miR-199a-3p	[171]
Cilostazol	Phosphodiesterase-3 (PDE3) inhibitor	STZ-induced diabetic rat	Sprague–Dawley rats	A high dose of cilostazol group (*n* = 13, 27 mg/kg/d), and a low dose of cilostazol group (*n* = 13, 9 mg/kg/d), for 8 weeks	↓ VCAM-1, ICAM-1, MCP-1 and VEGF, inactivation of NF-κB. ↓ glomerular hyperplasia, inflammatory cells infiltration and increased relative kidney weight	[172]
Cilostazol	Phosphodiesterase-3 (PDE3) inhibitor	High fat diet (HFD)/low-dose STZ-induced nephropathy	Mouse (C56BL/6 J)	Cilostazol (30 mg/kg), via oral gavage 5 days a week, for 13 weeks	↓ Mesangial expansion, vasodilated and vacuolated tubules. ↓ UACR and urinary cystatin C levels	[173]
Cilostazol	Phosphodiesterase-3 (PDE3) inhibitor	DKD in a STZ-induced T1DM. model	Sprague–Dawley rats	Cilostazol (5 mg/kg or 25 mg/kg) for 6 and 12 weeks	Beneficial effects of small dosage (5 mg/kg/d) of cilostazol on STZ-induced DKD from 6 weeks of treatment ↓ glomerular size, hydropic changes in PCT, and expansional mesangial matrix. ↓ albuminuria, total cholesterol, TG, and LDL-C ↓ ROS activity, NF-kB, and TGF-β ↓ MDA, ↑ catalase, GSH	[174]
Cilostazol	Phosphodiesterase-3 (PDE3) inhibitor	DKD in a STZ-induced T1DM. model	Sprague–Dawley rats	Cilostazol 5 mg/kg/day, for 6 and 12 weeks	↓ Thickness of the GBM and improved mitochondrial morphology in mesangial cells of diabetic kidney ↓ ROS activity, NF-kB, and TGF-β	[175]
Dilazep hydrochloride	Adenosine uptake inhibitor	Otsuka Long-Evans Tokushima fatty (OLETF) rats, a T2DM animal model	Rat	Not mentioned	↓ Increased urinary protein excretion and NAG activity (indicator of renal tubular dysfunction). Prevented glomerulosclerosis and tubular atrophy and ↓ type IV collagen in the glomeruli.	[176]
Dipyridamole (DIP)	Platelet cAMP-PDE inhibitor	STZ-induced insulin dependent diabetes mellitus (IDDM)	Sprague–Dawley rats	DIP (50 mg/100 g twice a day via a gastric tube)	↑ Activity of tubuloglomerular feedback, urinary protein excretion (UPE), GFR	[177]
Dipyridamole (DIP)	Platelet cAMP-PDE inhibitor	STZ-induced IDDM	Male Sprague–Dawley rats	DIP 6 mg/kg/day orally for 2 weeks.	↓ Serum creatinine, blood urea nitrogen levels, urinary albumin excretion ↓ MDA, ↑ SOD ↓ ICAM-1, TNF-α, IL-18 ↑ IL-10 ↓ activation of caspases-3/8/9 and pJNK/JNK Blocked diabetes-induced reduction in renal adenosine protein expression level. Developed mild glomerular affection and vacuolation of tubular epithelium	[178]
Ticagrelor	P2Y12 antagonists	Combined the STZ injections with unilateral nephrectomy-induced T1DM mouse model	Mouse (C57BL6/J)	Ticagrelor 300 mg/kg, oral gavage every other day, for 16 weeks	↓ Albuminuria, glomerular injury, endothelial cell activation and injury, and tubulointerstitial fibrosis, inflammation, and tubular apoptosis.	[179]
Beraprost	Analogue prostaglandin I_2_ (PGI_2_)	A high-fat diet–low dose STZ to establish the rat model of type 2 DKD	Male Sprague–Dawley rats	BPS given daily 0.6 mg/kg intragastric for 8 weeks of treatment	↓ Blood glucose, urine output, 24 h UAlb, Cr, hs-CRP, and IL-6 levels ↑ body mass	[180]
Beraprost	Analogue prostaglandin I_2_ (PGI_2_)	STZ-induced T1DM mouse model	Male Sprague–Dawley rats	BPS 30 mg per rat per day (250 mg/kg per day), intraperitoneal administration, for 28 days	↓ Creatinine clearance, albumin excretion, ICAM-1, macrophages, ecNOS in afferent arterioles and glomeruli, diameters afferent arterioles and glomeruli	[181]

**Table 4:** Antiplatelets drug therapy in human studies of diabetes kidney disease.

Therapeutic Intervention	Target of Action	Type of Patients	Number of Patients	Treatment Dose and Duration	Renal Effects	Ref.
Clopidogrel	inhibitor of the ADP P2Y12 receptor	Patients with DKD	*N* = 15,603	Clopidogrel 75 mg/day, followed up for a median of 28 months	Effect in kidney (-) Effect on cardiovascular event and mortality ↑	[182]
Sarpogrelate hydrochloride	5-hydroxytryptamine [5-HT_2_A] receptor antagonist	T2DM patients on metformin-based antidiabetic therapy	*N* = 478	Not specified	↓ The incidence and progression of nephropathy	[183]
Sarpogrelate hydrochloride	5-hydroxytryptamine [5-HT_2_A] receptor antagonist	Diabetic patients with nephropathy and arteriosclerosis obliterans and treated with ARB	*N* = 40	Sarpogrelate (300 mg/d)	↓ Albuminuria and plasma and urinary MCP-1 levels, ↑ plasma adiponectin	[184]
Sarpogrelate hydrochloride	5-hydroxytryptamine [5-HT_2_A] receptor antagonist	T2DM patients	*N* = 10	Sarpogrelate hydrochloride (200–300 mg/day)	↓ Urinary albumin excretion level	[185]
Cilostazol	Phosphodiesterase-3 (PDE3) inhibitor	T2DM patients with early nephropathy	*N* = 60	Cilostazol 100 mg in the morning and evening after meals, for 6 months.	↓ sICAM-1, MCP-1 and UAER levels No abnormal change in blood pressure, liver or kidney function, or HbA1c level	[168]
Cilostazol	Phosphodiesterase-3 (PDE3) inhibitor	Patient with DKD	*N* = 90	Cilostazol 100 mg, twice daily, for 52 weeks	↓ Microabuminuria, ACR (albumin to creatinine ratio ↓ E-selectin, MCP-1, sVCAM-1	[186]
Cilostazol	Phosphodiesterase-3 (PDE3) inhibitor	Patient T1DM with microalbuminuria	*N* = 13	Cilostazol 100 mh, daily, for 3 months	↓ Urinary albumin index, urinary TXB ↑ 6KF/TXB ratio No effects on blood glucose, HbA1bc and lipid	[187]
Beraprost and Cilostazol	Beraprost: Analogue prostaglandin I_2_ (PGI_2_)	NIDDM patients with nephropathy and chronic arterial obstruction	*N* = 98	Beraprost (60–120 µg/day) (*n* = 7) and cilostazol (100 mg/day) (*n* = 6), for 3 months	Beraprost: ↓ TM level by 1 month after the start of treatment. Cilostazol: a slight decrease of TM level at 1 month after treatment and a significant ↓ at 3 months after treatment.	[188]
Beraprost	Analogue prostaglandin I_2_ (PGI_2_)	T2DM patients with microalbuminuria	*N* = 52	Beraprost 20 μg/tablet, 2 tablets, three times a day (120 μg/day), for 24 weeks	↓ Microalbuminuria	[189]
Beraprost	Analogue prostaglandin I_2_ (PGI_2_)	Elderly patients with DKD	*N* = 100	Additional Beraprost sodium (20 μg/tablet, 2 tablets, three times a day) on the basis of the routine treatment, for 15 days	↓ Urinary protein, BUN, Scr and Cys-C	[190]
Beraprost	Analogue prostaglandin I_2_ (PGI_2_)	Japanese patients (age > 30 years) with DKD and arteriosclerosis obliterans	*N* = 26	Combination of an RAS inhibitor and BPS (120 μg/day), for 48 weeks	Serum creatinine, creatinine, cystatin C and the eGFR were unchanged	[191]
Beraprost combination	Analogue prostaglandin I_2_ (PGI_2_)	type 2 DKD	*N* = 102	Alprostadil 10 µg, for 2 weeks, followed by beraprost 40 µg twice a day. All patients continued treatment with conventional drugs (gliguidone or insulin, aspirin, simvastatin)	↓ Fasting blood glucose, blood viscosity, plasma viscosity and erythrocyte deformation exponent ↓ fibrinogen (FIB), D dimer and platelets ↓ UACR, CysC, β2-MG and α1-MG ↓ renin and angiotensin II and TNF-α	[192]
Dilazep hydrochloride	Adenosine uptake inhibitor	Microalbuminuria stage of DKD patient	*N* = 37	Dilazep hydrochloride 300 mg/day, orally, 6 months	↓ Albuminuria, prevents renal functional deterioration	[193]
Dilazep dihydrochloride	Adenosine uptake inhibitor	Patients with T2DM and microalbuminuria	*N* = 80	Dilazep dihydrochloride (300 mg/day; *n* = 9), for 6 months. Treatment only for diabetic patients positive for urinary podocytes	↓ Microalbuminuria and the number of urinary podocytes	[194]
Aspirin–dipyridamole	Dipyridamole inhibition of platelet cAMP-phosphodiesterase Aspirin block thromboxane A2	Type 2 DKD patients	*N* = 76	Aspirin (1000 mg) or Dipyridamole (750 mg), or their combination daily for 2 months	↓ Proteinuria, with the most prominent effect seen with combination of the 2 drugs	[195]
Aspirin–dipyridamole	Dipyridamole inhibition of platelet cAMP-phosphodiesterase Aspirin block thromboxane A2	Patient with IDDM with nephropathy	*N* = 16	Aspirin–dipyridamole 990 mg/225 mg daily; 6 weeks	↓ Urinary protein excretion	[196]

## References

[1] Lim A. (2014). Diabetic nephropathy–complications and treatment. Int. J. Nephrol. Renov. Dis..

[2] Young B.A., Johnson R.J., Alpers C.E., Eng E., Gordon K., Floege J., Couser W.G., Statistical Assistance from Seidel Kristy Seidel (1995). Cellular events in the evolution of experimental diabetic nephropathy. Kidney Int..

[3] Gheith O., Farouk N., Nampoory N., A Halim M., Al-Otaibi T. (2015). Diabetic kidney disease: World wide difference of prevalence and risk factors. J. Nephropharmacol..

[4] Alicic R.Z., Rooney M.T., Tuttle K.R. (2017). Diabetic Kidney Disease: Challenges, Progress, and Possibilities. Clin. J. Am. Soc. Nephrol..

[5] Ameh O.I., Okpechi I.G., Agyemang C., Kengne A.P., Roelofs J.J.T.H., Vogt L. (2019). Global, regional, and ethic differences in diabetic nephropathy. Diabetic Nephropathy: Pathophysiology and Clinical Aspects.

[6] CDC (2021). Chronic Kidney Disease in the United States.

[7] Kanasaki K., Taduri G., Koya D. (2013). Diabetic nephropathy: The role of inflammation in fibroblast activation and kidney fibrosis. Front. Endocrinol..

[8] Lim A.K.H., Tesch G.H. (2012). Inflammation in Diabetic Nephropathy. Mediat. Inflamm..

[9] IJpelaar D.H.T., Roelofs J.J.T.H., Vogt L. (2019). Inflammatory Process in Diabetic Glomeruli. Diabetic Nephropathy: Pathophysiology and Clinical Aspects.

[10] Boccardo P., Remuzzi G., Galbusera M. (2004). Platelet Dysfunction in Renal Failure. Semin. Thromb. Hemost..

[11] Sonmez O., Sonmez M. (2017). Role of platelets in immune system and inflammation. Porto Biomed. J..

[12] Carr M.E. (2001). Diabetes mellitus: A hypercoagulable state. J. Diabetes Its Complicat..

[13] Kaur R., Kaur M., Singh J. (2018). Endothelial dysfunction and platelet hyperactivity in type 2 diabetes mellitus: Molecular insights and therapeutic strategies. Cardiovasc. Diabetol..

[14] Ghoshal K., Bhattacharyya M. (2014). Overview of Platelet Physiology: Its Hemostatic and Nonhemostatic Role in Disease Pathogenesis. Sci. World J..

[15] Tervaert T.W.C., Mooyaart A.L., Amann K., Cohen A.H., Cook H.T., Drachenberg C.B., Ferrario F., Fogo A.B., Haas M., de Heer E. (2010). Pathologic Classification of Diabetic Nephropathy. J. Am. Soc. Nephrol..

[16] Najafian B., Alpers C.E., Roelofs J.J.T.H., Vogt L. (2019). Pathology of kidney in diabetes. Diabetic Nephropathy: Pathophysiology and Clinical Aspects.

[17] Reidy K., Kang H.M., Hostetter T., Susztak K. (2014). Molecular mechanisms of diabetic kidney disease. J. Clin. Investig..

[18] Kanwar Y.S., Sun L., Xie P., Liu F.-Y., Chen S. (2011). A Glimpse of Various Pathogenetic Mechanisms of Diabetic Nephropathy. Annu. Rev. Pathol. Mech. Dis..

[19] Chang J., Yan J., Li X., Liu N., Zheng R., Zhong Y. (2021). Update on the Mechanisms of Tubular Cell Injury in Diabetic Kidney Disease. Front. Med..

[20] Nowak N., Skupien J., Niewczas M.A., Yamanouchi M., Major M., Croall S., Smiles A., Warram J.H., Bonventre J.V., Krolewski A.S. (2016). Increased plasma kidney injury molecule-1 suggests early progressive renal decline in non-proteinuric patients with type 1 diabetes. Kidney Int..

[21] Qi R., Yang C. (2018). Renal tubular epithelial cells: The neglected mediator of tubulointerstitial fibrosis after injury. Cell Death Dis..

[22] Gilbert R.E. (2017). Proximal Tubulopathy: Prime Mover and Key Therapeutic Target in Diabetic Kidney Disease. Diabetes.

[23] Rossing P., Frimodt-Moller M., Roelofs J.J.T.H., Vogt L. (2019). Clinical features and natural course of diabetic nephropathy. Diabetic Nephropathy: Pathophysiology and Clinical Aspects.

[24] Haneda M., Utsunomiya K., Koya D., Babazono T., Moriya T., Makino H., Kimura K., Suzuki Y., Wada T., Ogawa S. (2014). A new classification of Diabetic Nephropathy 2014: A report from Joint Committee on Diabetic Nephropathy. Clin. Exp. Nephrol..

[25] Zheng W., Guo J., Liu Z.-S. (2021). Effects of metabolic memory on inflammation and fibrosis associated with diabetic kidney disease: An epigenetic perspective. Clin. Epigenet..

[26] Bohle A., Wehrmann M., Bogenschütz O., Batz C., Müller G. (1991). The Pathogenesis of Chronic Renal Failure in Diabetic Nephropathy: Investigation of 488 Cases of Diabetic Glomerulosclerosis. Pathol.-Res. Pract..

[27] Yiu W.H., Lin M., Tang S.C. (2014). Toll-like receptor activation: From renal inflammation to fibrosis. Kidney Int. Suppl..

[28] Garcia-Garcia P.M., Getino-Melian M.A., Dominguez-Pimentel V., Navarro-Gonzalez J.F. (2014). Inflammation in diabetic kidney disease. World J. Diabetes.

[29] Rivero A., Mora C., Muros M., García J., Herrera H., Navarro-González J. (2009). Pathogenic perspectives for the role of inflammation in diabetic nephropathy. Clin. Sci..

[30] El Hafez A.A. (2014). Microinflammation as a Candidate for Diabetic Nephropathy. Interdiscip. J. Microinflamm..

[31] Luis-Rodriguez D., Martinez-Castelao A., Gorriz J.L., De-Alvaro F., Navarro-Gonzalez J.F. (2012). Pathophysiological role and therapeutic implications of inflammation in diabetic nephropathy. World J. Diabetes.

[32] Duran-Salgado M.B., Rubio-Guerra A.F. (2014). Diabetic nephropathy and inflammation. World J. Diabetes.

[33] Mora C., Navarro J.F. (2004). Inflammation and pathogenesis of diabetic nephropathy. Metabolism.

[34] Nazir N., Siddiqui K., Al-Qasim S., Al-Naqeb D. (2014). Meta-analysis of diabetic nephropathy associated genetic variants in inflammation and angiogenesis involved in different biochemical pathways. BMC Med. Genet..

[35] Erem C., Hacıhasanoğlu A., Çelik Ş., Ovalı E., Ersöz H., Ukinç K., Deger O., Telatar M. (2004). Coagulation and Fibrinolysis Parameters in Type 2 Diabetic Patients with and without Diabetic Vascular Complications. Med. Princ. Pract..

[36] Roelofs J.J., Roelofs J.J., Vogt L. (2019). Coagulation and Hemostasis in Diabetic Nephropathy. Diabetic Nephropathy: Pathophysiology and Clinical Aspects.

[37] Sobel B.E., Schneider D.J. (2004). Platelet function, coagulopathy, and impaired fibrinolysis in diabetes. Cardiol. Clin..

[38] Astrug A., Khristov L., Stamenova T. (1975). Changes in blood coagulation in diabetic nephropathy. Vutreshni Boles..

[39] Elwakiel A., Shahzad K., Rana R., Gupta D., Gadi I., Kohli S., Renné T., Isermann B. (2020). Coagulation Factor XII Signaling Triggers Inflammasome-associated Renal Damage in Diabetic Nephropathy [abstract]. Res. Pract. Thromb. Haemost..

[40] Madan R., Gupt B., Saluja S., Kansra U.C., Tripathi B.K., Guliani B.P. (2010). Coagulation profile in diabetes and its association with diabetic microvascular complications. J. Assoc. Physicians India.

[41] Aso Y., Yoshida N., Okumura K.-I., Wakabayashi S., Matsutomo R., Takebayashi K., Inukai T. (2004). Coagulation and inflammation in overt diabetic nephropathy: Association with hyperhomocysteinemia. Clin. Chim. Acta.

[42] Mitsui S., Oe Y., Sekimoto A., Sato E., Hashizume Y., Yamakage S., Kumakura S., Sato H., Ito S., Takahashi N. (2020). Dual blockade of protease-activated receptor 1 and 2 additively ameliorates diabetic kidney disease. Am. J. Physiol. Physiol..

[43] Waasdorp M., Duitman J., Florquin S., Spek C.A. (2016). Protease-activated receptor-1 deficiency protects against streptozotocin-induced diabetic nephropathy in mice. Sci. Rep..

[44] Ito Y., Aten J., Bende R.J., Oemar B.S., Rabelink T.J., Weening J.J., Goldschmeding R. (1998). Expression of connective tissue growth factor in human renal fibrosis. Kidney Int..

[45] Tung C.-W., Hsu Y.-C., Shih Y.-H., Chang P.-J., Lin C.-L. (2018). Glomerular mesangial cell and podocyte injuries in diabetic nephropathy. Nephrology.

[46] Brosius F.C. (2008). New insights into the mechanisms of fibrosis and sclerosis in diabetic nephropathy. Rev. Endocr. Metab. Disord..

[47] Eitner F., Floege J. (2003). Novel insights into renal fibrosis. Curr. Opin. Nephrol. Hypertens..

[48] Vallon V., Komers R. (2011). Pathophysiology of the Diabetic Kidney. Compr. Physiol..

[49] El Mesallamy H.O., Ahmed H., Bassyouni A.A., Ahmed A.S. (2012). Clinical significance of inflammatory and fibrogenic cytokines in diabetic nephropathy. Clin. Biochem..

[50] Kolset S.O., Reinholt F.P., Jenssen T. (2012). Diabetic Nephropathy and Extracellular Matrix. J. Histochem. Cytochem..

[51] Garud M.S., Kulkarni Y.A. (2014). Hyperglycemia to nephropathy via transforming growth factor beta. Curr. Diabetes Rev..

[52] Kashihara N., Haruna Y., Kondeti V.K., Kanwar Y.S. (2010). Oxidative Stress in Diabetic Nephropathy. Curr. Med. Chem..

[53] Ferroni P., Martini F., Riondino S., La Farina F., Magnapera A., Ciatti F., Guadagni F. (2009). Soluble P-selectin as a marker of in vivo platelet activation. Clin. Chim. Acta.

[54] Sakharova O.V., Taal M., Brenner B.M. (2001). Pathogenesis of diabetic nephropathy: Focus on transforming growth factor-β and connective tissue growth factor. Curr. Opin. Nephrol. Hypertens..

[55] Van Roeyen C.R., Ostendorf T., Floege J. (2012). The platelet-derived growth factor system in renal disease: An emerging role of endogenous inhibitors. Eur. J. Cell Biol..

[56] Roestenberg P., van Nieuwenhoven F.A., Wieten L., Boer P., Diekman T., Tiller A.M., Wiersinga W.M., Oliver N., Usinger W., Weitz S. (2004). Connective Tissue Growth Factor Is Increased in Plasma of Type 1 Diabetic Patients With Nephropathy. Diabetes Care.

[57] Chiarelli F., Gaspari S., Marcovecchio M.L. (2009). Role of Growth Factors in Diabetic Kidney Disease. Horm. Metab. Res..

[58] Wang S.-N., Lapage J., Hirschberg R. (2000). Role of glomerular ultrafiltration of growth factors in progressive interstitial fibrosis in diabetic nephropathy. Kidney Int..

[59] Mason R.M., Wahab N.A. (2003). Extracellular Matrix Metabolism in Diabetic Nephropathy. J. Am. Soc. Nephrol..

[60] Floege J., Eng E., Young B.A., Alpers C.E., Barrett T.B., Bowen-Pope D.F., Johnson R.J. (1993). Infusion of platelet-derived growth factor or basic fibroblast growth factor induces selective glomerular mesangial cell proliferation and matrix accumulation in rats. J. Clin. Investig..

[61] Koszegi S., Molnar A., Lenart L., Hodrea J., Balogh D.B., Lakat T., Szkibinszkij E., Hosszu A., Sparding N., Genovese F. (2018). RAAS inhibitors directly reduce diabetes-induced renal fibrosis via growth factor inhibition. J. Physiol..

[62] Simonson M.S. (2007). Phenotypic transitions and fibrosis in diabetic nephropathy. Kidney Int..

[63] Zeisberg E.M., Potenta S.E., Sugimoto H., Zeisberg M., Kalluri R. (2008). Fibroblasts in kidney fibrosis emerge via endothelial-to-mesenchymal transition. J. Am. Soc. Nephrol..

[64] Lee S.B., Kalluri R. (2010). Mechanistic connection between inflammation and fibrosis. Kidney Int..

[65] Tanji N., Markowitz G.S., Fu C., Kislinger T., Taguchi A., Pischetsrieder M., Stern D., Schmidt A.M., D’Agati V.D. (2000). Expression of Advanced Glycation End Products and Their Cellular Receptor RAGE in Diabetic Nephropathy and Nondiabetic Renal Disease. J. Am. Soc. Nephrol..

[66] Wendt T.M., Tanji N., Guo J., Kislinger T.R., Qu W., Lu Y., Bucciarelli L.G., Rong L.L., Moser B., Markowitz G.S. (2003). RAGE Drives the Development of Glomerulosclerosis and Implicates Podocyte Activation in the Pathogenesis of Diabetic Nephropathy. Am. J. Pathol..

[67] Ribatti D., Crivellato E. (2007). Giulio Bizzozero and the discovery of platelets. Leuk. Res..

[68] Gazzaniga V., Ottini L. (2001). The discovery of platelets and their function. Vesalius.

[69] Twomey L., Wallace R.G., Cummins P.M., Degryse B., Sheridan S., Harrison M., Moyna N., Meade-Murphy G., Navasiolava N., Custaud M.-A., Lasakosvitsch F., Garnes S.D.A. (2019). Platelets: From Formation to Function. Homeostasis-An Integrated Vision.

[70] Yun S.-H., Sim E.-H., Goh R.-Y., Park J.-I., Han J.-Y. (2016). Platelet Activation: The Mechanisms and Potential Biomarkers. BioMed Res. Int..

[71] Chen J., Tan W. (2020). Platelet activation and immune response in diabetic microangiopathy. Clin. Chim. Acta.

[72] Drelich D.A., Bray P.F., Kerrigan S.W., Moran N. (2015). The traditional role of platelets in hemostasis. The Non-Thrombotic Role of Platelets in Health and Disease.

[73] White J.G., Michelson A.D. (2013). Platelet structure. Platelets.

[74] Duerschmied D., Massberg S., Zirlik A., Bode C., Gawaz M. (2017). Platelets as Regulators of Thrombosis and Inflammation. Platelets, Haemostasis and Inflammation. Cardiac and Vascular Biology.

[75] Koupenova M., Clancy L., Corkrey H.A., Freedman J.E. (2018). Circulating Platelets as Mediators of Immunity, Inflammation, and Thrombosis. Circ. Res..

[76] Spencer F.A., Becker R.C. (1997). Platelets: Structure, Function, and Their Fundamental Contribution to Hemostasis and Pathologic Thrombosis. Textbook of Coronary Thrombosis and Thrombolysis.

[77] Rasche H. (2001). Haemostasis and thrombosis: An overview. Eur. Heart J. Suppl..

[78] Broos K., Feys H.B., De Meyer S.F., Vanhoorelbeke K., Deckmyn H. (2011). Platelets at work in primary hemostasis. Blood Rev..

[79] Boon G.D. (1993). An overview of hemostasis. Toxicol. Pathol..

[80] Holinstat M. (2017). Normal platelet function. Cancer Metastasis Rev..

[81] Hou Y., Carrim N., Wang Y., Gallant R.C., Marshall A., Ni H. (2015). Platelets in hemostasis and thrombosis: Novel mechanisms of fibrinogen-independent platelet aggregation and fibronectin mediated protein wave of hemostasis. J. Biomed. Res..

[82] Barale C., Russo I. (2020). Influence of Cardiometabolic Risk Factors on Platelet Function. Int. J. Mol. Sci..

[83] Lim H.S., Blann A.D., Lip G.Y. (2004). Soluble CD40 ligand, soluble P-selectin, interleukin-6, and tissue factor in diabetes mellitus: Relationships to cardiovascular disease and risk factor intervention. Circulation.

[84] Kakouros N., Rade J.J., Kourliouros A., Resar J.R. (2011). Platelet Function in Patients with Diabetes Mellitus: From a Theoretical to a Practical Perspective. Int. J. Endocrinol..

[85] Kubisz P., Stančiaková L., Staško J., Galajda P., Mokáň M. (2015). Endothelial and platelet markers in diabetes mellitus type 2. World J. Diabetes.

[86] Ferroni P., Basili S., Falco A., Davì G. (2004). Platelet activation in type 2 diabetes mellitus. J. Thromb. Haemost..

[87] Turgutalp K., Ozhan O., Akbay E., Tombak A., Tiftik N., Ozcan T., Yilmaz S., Helvaci I., Kiykim A. (2014). Mean platelet volume and related factors in patients at different stages of diabetic nephropathy: A preliminary study. Clin. Appl. Thromb. Hemost..

[88] Bavbek N., Kargili A., Kaftan O., Karakurt F., Kosar A., Akcay A. (2007). Elevated concentrations of soluble adhesion molecules and large platelets in diabetic patients: Are they markers of vascular disease and diabetic nephropathy?. Clin. Appl. Thromb. Hemost..

[89] Niewiarowski S., Thomas D.P. (1969). Platelet Factor 4 and Adenosine Diphosphate Release during Human Platelet Aggregation. Nature.

[90] Manne B.K., Xiang S.C., Rondina M.T. (2016). Platelet secretion in inflammatory and infectious diseases. Platelets.

[91] Eisman R., Surrey S., Ramachandran B., Schwartz E., Poncz M. (1990). Structural and functional comparison of the genes for human platelet factor 4 and PF4alt. Blood.

[92] Kowalska M.A., Rauova L., Poncz M. (2010). Role of the platelet chemokine platelet factor 4 (PF4) in hemostasis and thrombosis. Thromb. Res..

[93] Cella G., Scattolo N., Girolami A., Sasahara A.A. (1984). Are platelet factor 4 and β-thromboglobulin markers of cardiovascular disorders?. Ric. Clin. E Lab..

[94] Gurney D., Lip G.Y., Blann A.D. (2002). A reliable plasma marker of platelet activation: Does it exist?. Am. J. Hematol..

[95] Gruden G., Cavallo-Perin P., Romagnoli R., Ruiu G., Pagano G. (1994). Plasma beta-thromboglobulin and platelet factor 4 are not increased in insulin-dependent diabetic patients with microalbuminuria. Geol. Rundsch..

[96] Hopper A.H., Tindall H., A Davies J. (1986). Urinary beta-thromboglobulin correlates with impairment of renal function in patients with diabetic nephropathy. Thromb. Haemost..

[97] Endo Y., Mamiya S., Satoh M., Takahashi K., Harada T. (1981). Plasma β-thromboglobulin and platelet factor 4 in patients with chronic renal failure and effect of hemodialysis. Tohoku J. Exp. Med..

[98] Andrassy K., Deppermann D., Ritz E., Koderisch J., Seelig H. (1980). Different effects of renal failure on beta-thromboglobulin and high affinity platelet factor 4 (HA-PF4)-concentrations. Thromb. Res..

[99] Appay V., Rowland-Jones S.L. (2001). RANTES: A versatile and controversial chemokine. Trends Immunol..

[100] Pichler R., Afkarian M., Dieter B.P., Tuttle K.R. (2017). Immunity and inflammation in diabetic kidney disease: Translating mechanisms to biomarkers and treatment targets. Am. J. Physiol. Physiol..

[101] Mezzano S., Aros C., Droguett A., Burgos M.E., Ardiles L., Flores C., Schneider H., Ruiz-Ortega M., Egido J. (2004). NF-kappaB activation and overexpression of regulated genes in human diabetic nephropathy. Nephrol. Dial. Transplant..

[102] Flaumenhaft R., Michelson A.D. (2013). Chapter 18—Platelet Secretion. Platelets.

[103] Floege J., Eitner F., Alpers C.E. (2007). A New Look at Platelet-Derived Growth Factor in Renal Disease. J. Am. Soc. Nephrol..

[104] Schrijvers B.F., De Vriese A., Flyvbjerg A. (2004). From Hyperglycemia to Diabetic Kidney Disease: The Role of Metabolic, Hemodynamic, Intracellular Factors and Growth Factors/Cytokines. Endocr. Rev..

[105] Johnson R., Iida H., Yoshimura A., Floege J., Bowen-Pope D.F. (1992). Platelet-derived growth factor: A potentially important cytokine in glomerular disease. Kidney Int..

[106] Johnson R.J., Floege J., Couser W.G., Alpers C.E. (1993). Role of platelet-derived growth factor in glomerular disease. J. Am. Soc. Nephrol..

[107] Riley S.G., Steadman R., Williams J.D., Floege J., Phillips A.O. (1999). Augmentation of kidney injury by basic fibroblast growth factor or platelet-derived growth factor does not induce progressive diabetic nephropathy in the Goto Kakizaki model of non-insulin-dependent diabetes. J. Lab. Clin. Med..

[108] Boor P., Bábíčková J., Steegh F., Hautvast P., Martin I.V., Djudjaj S., Nakagawa T., Ehling J., Gremse F., Bücher E. (2015). Role of Platelet-Derived Growth Factor-CC in Capillary Rarefaction in Renal Fibrosis. Am. J. Pathol..

[109] Abboud H.E. (1992). Platelet-derived growth factor and mesangial cells. Kidney Int..

[110] Nakagawa T., Inoue H., Sasahara M. (2012). Platelet-derived growth factor and renal disease. Curr. Opin. Nephrol. Hypertens..

[111] Suzuki H., Usui I., Kato I., Oya T., Kanatani Y., Yamazaki Y., Fujisaka S., Senda S., Ishii Y., Urakaze M. (2011). Deletion of platelet-derived growth factor receptor-beta improves diabetic nephropathy in Ca^2+^/calmodulin-dependent protein kinase IIalpha (Thr286Asp) transgenic mice. Diabetologia.

[112] Langham R.G., Kelly D.J., Maguire J., Dowling J.P., Gilbert R.E., Thomson N.M. (2003). Over-expression of platelet-derived growth factor in human diabetic nephropathy. Nephrol. Dial. Transplant..

[113] Chen Y., Zhong H., Zhao Y., Luo X., Gao W. (2020). Role of platelet biomarkers in inflammatory response. Biomark. Res..

[114] André P., Nannizzi-Alaimo L., Prasad S.K., Phillips D.R. (2002). Platelet-Derived CD40L. Circulation.

[115] van Kooten C., Banchereau J. (2000). CD40-CD40 ligand. J. Leukoc. Biol..

[116] Henn V., Steinbach S., Büchner K., Presek P., Kroczek R.A. (2001). The inflammatory action of CD40 ligand (CD154) expressed on activated human platelets is temporally limited by coexpressed CD40. Blood.

[117] Inwald D.P., McDowall A., Peters M.J., Callard R.E., Klein N.J. (2003). CD40 Is Constitutively Expressed on Platelets and Provides a Novel Mechanism for Platelet Activation. Circ. Res..

[118] Furman M.I., Krueger L.A., Linden M.D., Barnard M.R., Frelinger A.L., Michelson A.D. (2004). Release of soluble CD40L from platelets is regulated by glycoprotein IIb/IIIa and actin polymerization. J. Am. Coll. Cardiol..

[119] Varo N., Libby P., Nuzzo R., Italiano J., Doria A., Schönbeck U. (2005). Elevated release of sCD40L from platelets of diabetic patients by thrombin, glucose and advanced glycation end products. Diabetes Vasc. Dis. Res..

[120] Lajer M., Tarnow I., Michelson A.D., Jorsal A., Frelinger A.L., Parving H.-H., Rossing P., Tarnow L. (2010). Soluble CD40 ligand is elevated in Type 1 diabetic nephropathy but not predictive of mortality, cardiovascular events or kidney function. Platelets.

[121] Chiarelli F., Giannini C., Verrotti A., Mezzetti A., Mohn A. (2008). Increased concentrations of soluble CD40 ligand may help to identify type 1 diabetic adolescents and young adults at risk for developing persistent microalbuminuria. Diabetes/Metabolism Res. Rev..

[122] Rigothier C., Daculsi R., Lepreux S., Auguste P., Villeneuve J., Dewitte A., Doudnikoff E., Saleem M., Bourget C., Combe C. (2016). CD154 Induces Matrix Metalloproteinase-9 Secretion in Human Podocytes. J. Cell. Biochem..

[123] Uwaezuoke S.N. (2019). The role of adhesion molecules in nephropathies: The diagnostic applications. Integr. Mol. Med..

[124] Avei E., Uzeli S. (2016). The role of adhesion molecules and cytokines in patients with diabetic nephropathy. Biomed. Res..

[125] Krieglstein C.F., Granger D.N. (2001). Adhesion molecules and their role in vascular disease. Am. J. Hypertens..

[126] Omoto S., Nomura S., Shouzu A., Hayakawa T., Shimizu H., Miyake Y., Yonemoto T., Nishikawa M., Fukuhara S., Inada M. (1999). Significance of Platelet-Derived Microparticles and Activated Platelets in Diabetic Nephropathy. Nephron Exp. Nephrol..

[127] Hirata K., Shikata K., Matsuda M., Akiyama K., Sugimoto H., Kushiro M., Makino H. (1998). Increased expression of selectins in kidneys of patients with diabetic nephropathy. Diabetologia.

[128] Théorêt J.-F., Yacoub D., Hachem A., Gillis M.-A., Merhi Y. (2011). P-selectin ligation induces platelet activation and enhances microaggregate and thrombus formation. Thromb. Res..

[129] Rossaint J., Zarbock A. (2015). Platelets in leucocyte recruitment and function. Cardiovasc. Res..

[130] Merten M., Thiagarajan P. (2000). P-Selectin Expression on Platelets Determines Size and Stability of Platelet Aggregates. Circulation.

[131] Xu L., Zhang Y., Chen J., Xu Y. (2020). Thrombospondin-1: A Key Protein That Induces Fibrosis in Diabetic Complications. J. Diabetes Res..

[132] Hugo C. (2003). The thrombospondin 1-TGF-beta axis in fibrotic renal disease. Nephrol. Dial. Transplant..

[133] Leung L.L. (1984). Role of thrombospondin in platelet aggregation. J. Clin. Investig..

[134] Dorahy D.J., Thorne R.F., Fecondo J.V., Burns G.F. (1997). Stimulation of Platelet Activation and Aggregation by a Carboxyl-terminal Peptide from Thrombospondin Binding to the Integrin-associated Protein Receptor. J. Biol. Chem..

[135] Abdelwhab S., Fooda O., Abdelmaksoud S. (2010). Thrombospondin-1 in Patients with Diabetic Nephropathy. Kidney.

[136] Vanhoutte D., Heymans S. (2011). Thrombospondin 1. Hypertension.

[137] Daniel C., Schaub K., Amann K., Lawler J., Hugo C. (2007). Thrombospondin-1 Is an Endogenous Activator of TGF-β in Experimental Diabetic Nephropathy In Vivo. Diabetes.

[138] Jansen M.P.B., Florquin S., Roelofs J.J.T.H. (2018). The role of platelets in acute kidney injury. Nat. Rev. Nephrol..

[139] A Ali R., Wuescher L.M., Worth R.G. (2015). Platelets: Essential components of the immune system. Curr. Trends Immunol..

[140] Gaertner F., Massberg S. (2019). Patrolling the vascular borders: Platelets in immunity to infection and cancer. Nat. Rev. Immunol..

[141] Ribeiro L.S., Branco L.M., Franklin B.S. (2019). Regulation of Innate Immune Responses by Platelets. Front. Immunol..

[142] Carestia A., Kaufman T., Schattner M. (2016). Platelets: New Bricks in the Building of Neutrophil Extracellular Traps. Front. Immunol..

[143] Jansen M.P., Emal D., Teske G.J., Dessing M.C., Florquin S., Roelofs J.J. (2016). Release of extracellular DNA influences renal ischemia reperfusion injury by platelet activation and formation of neutrophil extracellular traps. Kidney Int..

[144] Salazar-Gonzalez H., Zepeda-Hernandez A., Melo Z., Saavedra-Mayorga D.E., Echavarria R. (2019). Neutrophil Extracellular Traps in the Establishment and Progression of Renal Diseases. Medicina.

[145] Miyoshi A., Yamada M., Shida H., Nakazawa D., Kusunoki Y., Nakamura A., Tomaru U., Atsumi T., Ishizu A. (2016). Supplementary Material for: Circulating Neutrophil Extracellular Trap Levels in Well-Controlled Type 2 Diabetes and Pathway Involved in Their Formation Induced by High-Dose Glucose. Pathobiology.

[146] Wang L., Zhou X., Yin Y., Mai Y., Wang D., Zhang X. (2019). Hyperglycemia Induces Neutrophil Extracellular Traps Formation Through an NADPH Oxidase-Dependent Pathway in Diabetic Retinopathy. Front. Immunol..

[147] Gómez R.M., Ortiz A.O.L., Schattner M. (2020). Platelets and extracellular traps in infections. Platelets.

[148] Okubo K., Kurosawa M., Kamiya M., Urano Y., Suzuki A., Yamamoto K., Hase K., Homma K., Sasaki J., Miyauchi H. (2018). Macrophage extracellular trap formation promoted by platelet activation is a key mediator of rhabdomyolysis-induced acute kidney injury. Nat. Med..

[149] Lu C.C., Ma K.L., Ruan X.Z., Liu B.C. (2017). The Emerging Roles of Microparticles in Diabetic Nephropathy. Int. J. Biol. Sci..

[150] Rodrigues K.F., Pietrani N.T., Fernandes A.P., Bosco A.A., de Sousa M.C.R., Silva I.D.F.O., Silveira J.N., Campos F.M.F., Gomes K.B. (2018). Circulating microparticles levels are increased in patients with diabetic kidney disease: A case-control research. Clin. Chim. Acta.

[151] Zhang Y., Ma K.L., Gong Y.X., Wang G.H., Hu Z.B., Liu L., Lu J., Chen P.P., Lu C.C., Ruan X.Z. (2018). Platelet Microparticles Mediate Glomerular Endothelial Injury in Early Diabetic Nephropathy. J. Am. Soc. Nephrol..

[152] Uil M., Hau C.M., Ahdi M., Mills J.D., Kers J., Saleem M.A., Florquin S., Gerdes V.E.A., Nieuwland R., Roelofs J.J.T.H. (2019). Cellular origin and microRNA profiles of circulating extracellular vesicles in different stages of diabetic nephropathy. Clin. Kidney J..

[153] Yu M., Xie R., Zhang Y., Liang H., Hou L., Yu C., Zhang J., Dong Z., Tian Y., Bi Y. (2018). Phosphatidylserine on microparticles and associated cells contributes to the hypercoagulable state in diabetic kidney disease. Nephrol. Dial. Transplant..

[154] Lu G.-Y., Xu R.-J., Zhang S.-H., Qiao Q., Shen L., Li M., Xu D.-Y., Wang Z.-Y. (2015). Alteration of circulatory platelet microparticles and endothelial microparticles in patients with chronic kidney disease. Int. J. Clin. Exp. Med..

[155] Okumura M., Imanishi M., Okamura M., Hosoi M., Okada N., Konishi Y., Morikawa T., Miura K., Nakatani T., Fujii S. (2003). Role for thromboxane A2 from glomerular thrombi in nephropathy with type 2 diabetic rats. Life Sci..

[156] Thomas D.W., Coffman T.M. (1998). A genetic approach for studying the role of thromboxane A2 in the kidney. Kidney Int..

[157] Zoja C., Perico N., Bergamelli A., Pasini M., Morigi M., Dadan J., Belloni A., Bertani T., Remuzzi G. (1990). Ticlopidine prevents renal disease progression in rats with reduced renal mass. Kidney Int..

[158] Chignard M., Le Couedic J.P., Tence M., Vargaftig B.B., Benveniste J., Chignard J.P.L.C.M. (1979). The role of platelet-activating factor in platelet aggregation. Nature.

[159] Correa-Costa M., Andrade-Oliveira V., Braga T.T., Castoldi A., Aguiar C.F., Origassa C.S., Rodas A.C., Hiyane M.I., MAC Malheiros D., Rios F.J. (2014). Activation of platelet-activating factor receptor exacerbates renal inflammation and promotes fibrosis. Lab. Investig..

[160] Cavallo-Perin P., Lupia E., Gruden G., Olivetti C., De Martino A., Cassader M., Furlani D., Servillo L., Quagliuolo L., Iorio E. (2000). Increased blood levels of platelet-activating factor in insulin-dependent diabetic patients with microalbuminuria. Nephrol. Dial. Transplant..

[161] Perico N., Remuzzi A., Dadan J., Battaglia C., Remuzzi G. (1991). Platelet-activating factor alters glomerular barrier size selectivity for macromolecules in rats. Am. J. Physiol. Physiol..

[162] Zhou S., Huo D., He X., Yu P., Xiao Y., Ou C., Jiang R.-M., Li D., Li H. (2017). High glucose/lysophosphatidylcholine levels stimulate extracellular matrix deposition in diabetic nephropathy via platelet-activating factor receptor. Mol. Med. Rep..

[163] Rwibasira Rudinga G., Khan G.J., Kong Y. (2018). Protease-Activated Receptor 4 (PAR4): A Promising target for antiplatelet therapy. Int. J. Mol. Sci..

[164] Kahn M.L., Nakanishi-Matsui M., Shapiro M.J., Ishihara H., Coughlin S.R. (1999). Protease-activated receptors 1 and 4 mediate activation of human platelets by thrombin. J. Clin. Investig..

[165] Jansen M.P.B., Claessen N., Larsen P.W.B., Butter L.M., Florquin S., Roelofs J.J.T.H. (2019). Dual role of protease activated receptor 4 in acute kidney injury: Contributing to renal injury and inflammation, while maintaining the renal filtration barrier upon acute renal ischemia reperfusion injury. bioRxiv.

[166] Gremmel T., Kopp C.W., Seidinger D., Koppensteiner R., Steiner S., Panzer S. (2013). Impact of diabetes on platelet activation in different manifestations of atherosclerosis. Swiss Med. Wkly..

[167] Giannella A., Ceolotto G., Radu C.M., Cattelan A., Iori E., Benetti A., Fabris F., Simioni P., Avogaro A., Vigili de Kreutzenberg S. (2021). PAR-4/Ca^2+^-calpain pathway activation stimulates platelet-derived microparticles in hyperglycemic type 2 diabetes. Cardiovasc. Diabetol..

[168] Jiao X.-M., Jiao X.-J., Zhang X.-G., Xu X.-P., Wu J.-X., Yao L., Zhao J., Lü X.-F. (2013). Cilostazol reduces microalbuminuria in type 2 diabetic nephropathy. Chin. Med. J..

[169] Zheng Z., Ma T., Lian X., Gao J., Wang W., Weng W., Lu X., Sun W., Cheng Y., Fu Y. (2019). Clopidogrel Reduces Fibronectin Accumulation and Improves Diabetes-Induced Renal Fibrosis. Int. J. Biol. Sci..

[170] Lee E.Y., Lee M.Y., Kwon M.-H., Kim H.M., Kang J.S., Kim Y.M., Chung C.H. (2017). Sarpogrelate hydrochloride ameliorates diabetic nephropathy associated with inhibition of macrophage activity and inflammatory reaction in db/db mice. PLoS ONE.

[171] Kim S.K., Kim G., Choi B.-H., Ryu D., Ku S.-K., Kwak M.-K. (2020). Negative correlation of urinary miR-199a-3p level with ameliorating effects of sarpogrelate and cilostazol in hypertensive diabetic nephropathy. Biochem. Pharmacol..

[172] Wang F., Li M., Cheng L., Zhang T., Hu J., Cao M., Zhao J., Guo R., Gao L., Zhang X. (2008). Intervention with cilostazol attenuates renal inflammation in streptozotocin-induced diabetic rats. Life Sci..

[173] Park J.-H., Choi B.-H., Ku S.-K., Kim D.-H., Jung K.-A., Oh E., Kwak M.-K. (2017). Amelioration of high fat diet-induced nephropathy by cilostazol and rosuvastatin. Arch. Pharmacal Res..

[174] Lee W.C., Chen H.C., Wang C.Y., Lin P.Y., Ou T.T., Chen C.C., Wen M.C., Wang J., Lee H.J. (2010). Cilostazol ameliorates nephropathy in type 1 diabetic rats involving improvement in oxidative stress and regulation of TGF-Beta and NF-kappaB. Biosci. Biotechnol. Biochem..

[175] Chian C.-W., Lee Y.-S., Lee Y.-J., Chen Y.-H., Wang C.-P., Lee W.-C., Lee H.-J. (2020). Cilostazol ameliorates diabetic nephropathy by inhibiting highglucose- induced apoptosis. Korean J. Physiol. Pharmacol..

[176] Yamagishi S., Koga K., Inagaki Y., Amano S., Okamoto T., Takeuchi M. (2002). Dilazep hydrochloride, an antiplatelet drug, prevents progression of diabetic nephropathy in Otsuka Long-Evans Tokushima fatty rats [abstract]. Drugs Under Exp. Clin. Res..

[177] Vallon V., Osswald H. (1994). Dipyridamole prevents diabetes-induced alterations of kidney function in rats. Naunyn-Schmiedebergs Arch. fur Exp. Pathol. Pharmakol..

[178] Elsherbiny N., Al-Gayyar M.M., El Galil K.H.A. (2015). Nephroprotective role of dipyridamole in diabetic nephropathy: Effect on inflammation and apoptosis. Life Sci..

[179] Uil M., Butter L.M., Claessen N., Larsen P.W., Florquin S., Roelofs J.J.T.H. (2020). Platelet inhibition by ticagrelor is protective against diabetic nephropathy in mice. FASEB J..

[180] Guan J., Long L., Chen Y.-Q., Yin Y., Li L., Zhang C.-X., Deng L., Tian L.-H. (2014). Effects of beraprost sodium on renal function and inflammatory factors of rats with diabetic nephropathy. Genet. Mol. Res..

[181] Yamashita T., Shikata K., Matsuda M., Okada S., Ogawa D., Sugimoto H., Wada J., Makino H. (2002). Beraprost sodium, prostacyclin analogue, attenuates glomerular hyperfiltration and glomerular macrophage infiltration by modulating ecNOS expression in diabetic rats. Diabetes Res. Clin. Pract..

[182] Dasgupta A., Steinhubl S.R., Bhatt D.L., Berger P.B., Shao M., Mak K.-H., Fox K.A., Montalescot G., Weber M.A., Haffner S.M. (2009). Clinical Outcomes of Patients With Diabetic Nephropathy Randomized to Clopidogrel Plus Aspirin Versus Aspirin Alone (A post hoc Analysis of the Clopidogrel for High Atherothrombotic Risk and Ischemic Stabilization, Management, and Avoidance [CHARISMA] Trial). Am. J. Cardiol..

[183] Yoo H., Park I., Kim D.J., Lee S. (2019). Effects of sarpogrelate on microvascular complications with type 2 diabetes. Int. J. Clin. Pharm..

[184] Ogawa S., Mori T., Nako K., Ishizuka T., Ito S. (2008). Reduced Albuminuria with Sarpogrelate Is Accompanied by a Decrease in Monocyte Chemoattractant Protein-1 Levels in Type 2 Diabetes. Clin. J. Am. Soc. Nephrol..

[185] Takahashi T., Yano M., Minami J., Haraguchi T., Koga N., Higashi K., Kobori S. (2002). Sarpogrelate hydrochloride, a serotonin2A receptor antagonist, reduces albuminuria in diabetic patients with early-stage diabetic nephropathy. Diabetes Res. Clin. Pract..

[186] Tang W.-H., Lin F.-H., Lee C.-H., Kuo F.-C., Hsieh C.-H., Hsiao F.-C., Hung Y.-J. (2013). Cilostazol effectively attenuates deterioration of albuminuria in patients with type 2 diabetes: A randomized, placebo-controlled trial. Endocrine.

[187] Watanabe J., Sako Y., Umeda F., Nawata H. (1993). Effects of cilostazol, a phosphodiesterase inhibitor, on urinary excretion of albumin and prostaglandins in non-insulin-dependent diabetic patients. Diabetes Res. Clin. Pract..

[188] Hayakawa T., Shouzu A., Nishikawa M., Miyake Y., Shimizu H., Omoto S., Inada M. (2000). Effects of Beraprost and Cilostazol and Renal Function on Serum Thrombomodulin Levels in Diabetic Patients. Arzneimittelforschung.

[189] Choi Y.M., Kwon H.-S., Choi K.M., Lee W.-Y., Hong E.-G. (2019). Short-Term Effects of Beraprost Sodium on the Markers for Cardiovascular Risk Prediction in Type 2 Diabetic Patients with Microalbuminuria. Endocrinol. Metab..

[190] Xia J., Shen S. (2019). In Efficacy of beraprost sodium in the treatment of diabetic nephropathy in elderly patients. Int. J. Clin. Exp. Med..

[191] Shima A., Miyamoto M., Kubota Y., Takagi G., Shimizu W. (2015). Beraprost sodium protects against diabetic nephropathy in patients with arteriosclerosis obliterans: A prospective, randomized, open-label study. J. Nippon Med. Sch..

[192] Xu X., Pan X., Li S. (2019). Prospective analysis of the efficacy of beraprost sodium combined with alprostadil on diabetic nephropathy and influence on rennin-angiotensin system and TNF-α. Exp. Ther. Med..

[193] Koide H., Totsuka Y., Sugisaki T., Kitajima T., Ohmori Y., Kuriyama S., Oi K., Isogai S., Ohi H., Tomino Y. (1995). Clinical effect of the anti-platelet drug, dilazep dihydrochloride, in patients at the microalbuminuric stage of diabetic nephropathy—A multi-center study. Nihon Jinzo Gakkai Shi.

[194] Nakamura T., Ushiyama C., Shimada N., Sekizuka K., Ebihara I., Hara M., Koide H. (2000). Effect of the antiplatelet drug dilazep dihydrochloride on urinary podocytes in patients in the early stage of diabetic nephropathy. Diabetes Care.

[195] Khajehdehi P., Roozbeh J., Mostafavi H. (2002). A Comparative Randomized and Placebo-controlled Short-term Trial of Aspirin and Dipyridamole for Overt Type-2 Diabetic Nephropathy. Scand. J. Urol. Nephrol..

[196] Hopper A.H., Tindall H., Davies J.A. (1989). Administration of Aspirin-Dipyridamole Reduces Proteinuria in Diabetic Nephropathy. Nephrol. Dial. Transplant..

[197] Angiolillo D.J., Fernandez-Ortiz A., Bernardo E., Ramírez C., Sabaté M., Jimenez-Quevedo P., Hernández R., Moreno R., Escaned J., Alfonso F. (2006). Clopidogrel Withdrawal Is Associated With Proinflammatory and Prothrombotic Effects in Patients With Diabetes and Coronary Artery Disease. Diabetes.

[198] Yang Y., Kong D., Wang C., Chen G., Shan F., Qi K., Ma L. (2014). Inhibition of platelet activation could decrease thrombotic events in hemodialysis PF4/H antibody-positive patients. Ren. Fail..

[199] Liang L.-R., Ma Q., Feng L., Qiu Q., Zheng W., Xie W.-X. (2020). Long-term effect of clopidogrel in patients with and without diabetes: A systematic review and meta-analysis of randomized controlled trials. World J. Diabetes.

[200] Teng R. (2012). Pharmacokinetic, Pharmacodynamic and Pharmacogenetic Profile of the Oral Antiplatelet Agent Ticagrelor. Clin. Pharmacokinet..

[201] Dobesh P.P., Oestreich J. (2014). Ticagrelor: Pharmacokinetics, Pharmacodynamics, Clinical Efficacy, and Safety. Pharmacother. J. Hum. Pharmacol. Drug Ther..

[202] Nardin M., Verdoia M., Sartori C., Pergolini P., Rolla R., Barbieri L., Schaffer A., Bellomo G., Suryapranata H., De Luca G. (2016). Diabetes mellitus, glucose control parameters and platelet reactivity in ticagrelor treated patients. Thromb. Res..

[203] Alexopoulos D., Xanthopoulou I., Storey R.F., Bliden K.P., Tantry U.S., Angiolillo D.J., Gurbel P.A. (2014). Platelet reactivity during ticagrelor maintenance therapy: A patient-level data meta-analysis. Am. Heart J..

[204] Alexopoulos D., Vogiatzi C., Stavrou K., Vlassopoulou N., Perperis A., Pentara I., Xanthopoulou I. (2015). Diabetes mellitus and platelet reactivity in patients under prasugrel or ticagrelor treatment: An observational study. Cardiovasc. Diabetol..

[205] James S., Angiolillo D.J., Cornel J., Erlinge D., Husted S., Kontny F., Maya J., Nicolau J., Spinar J., Storey R. (2010). Ticagrelor vs. clopidogrel in patients with acute coronary syndromes and diabetes: A substudy from the PLATelet inhibition and patient Outcomes (PLATO) trial. Eur. Heart J..

[206] Malyszko J., Urano T., Knofler R., Taminato A., Yoshimi T., Takada Y., Takada A. (1994). Daily variations of platelet aggregation in relation to blood and plasma serotonin in diabetes. Thromb. Res..

[207] Małyszko J., Myśliwiec M. (1995). Blood platelet function, plasma serotonin and lipid metabolism in patients with diabetic nephropathy. Pol. Arch. Intern. Med..

[208] Park S.Y., Rhee S.Y., Oh S., Kwon H.-S., Cha B.-Y., Lee H.J., Kim Y.S. (2012). Evaluation of the effectiveness of sarpogrelate on the surrogate markers for macrovascular complications in patients with type 2 diabetes. Endocr. J..

[209] Kabil S.L. (2021). Cilostazol attenuates diabetic nephropathy in rats via elevation of plasma soluble receptor for advances glycation end-products. Al-Azhar J. Pharm. Sci..

[210] Asal N.J., Wojciak K.A. (2017). Effect of cilostazol in treating diabetes-associated microvascular complications. Endocrine.

[211] Sharma A.K., Khanna D., Balakumar P. (2014). Low-dose dipyridamole treatment partially prevents diabetes mellitus-induced vascular endothelial and renal abnormalities in rats. Int. J. Cardiol..

[212] Skálová S. (2005). The diagnostic role of urinary N-acetyl-beta-D-glucosaminidase (NAG) activity in the detection of renal tubular impairment. Acta Med..

[213] Yamamoto M., Fukui M., Kuramoto T., Kabuki K., Tomino Y. (1995). Effects of antiplatelet drug dilazep dihydrochloride on anionic sites and extracellular matrix (ecm) components in glomerular basement membrane of stz-induced diabetic rats. J. Clin. Lab. Anal..

[214] Umetsu T., Murata T., Nishio S. (1989). Studies on the antiplatelet effect of the stable epoprostenol analogue beraprost sodium and its mechanism of action in rats. Arzneimittelforschung.

[215] Melian E.B., Goa K.L. (2002). Beraprost: A review of its pharmacology and therapeutic efficacy in the treatment of peripheral arterial disease and pulmonary arterial hypertension. Drugs.

[216] Nony P., Ffrench P., Girard P., Delair S., Azoulay S., Girre J.P., Dechavanne M., Boissel J.P. (1996). Platelet-aggregation inhibition and hemodynamic effects of beraprost sodium, a new oral prostacyclin derivative: A study in healthy male subjects. Can. J. Physiol. Pharmacol..

[217] Chen S., Xie S., He W., Wei D., Li S., Chen W. (2017). Beneficial Effect of Beraprost Sodium Plus Aspirin in the Treatment of Acute Ischemic Stroke. Med. Sci. Monit..

[218] Mie K., Rie I., Teruhiko U., Masakazu H., Shintaro N. (1990). Prostacyclin and beraprost sodium as suppressors of activated rat polymorphonuclear leukocytes. Biochem. Pharmacol..

